# Evaluation of familial phenotype deviation to measure the impact of de novo mutations in autism

**DOI:** 10.1186/s13073-025-01532-7

**Published:** 2025-08-20

**Authors:** Soo-Whee Kim, Hyeji Lee, Da Yea Song, Gang-Hee Lee, Jae Hyun Han, Jee Won Lee, Hee Jung Byun, Ji Hyun Son, Ye Rim Kim, Yoojeong Lee, Eunjoon Kim, Donna M. Werling, So Hyun Kim, Stephan J. Sanders, Hee Jeong Yoo, Joon-Yong An

**Affiliations:** 1 Department of Integrated Biomedical and Life Science, Korea University, Seoul, Republic of Korea; 2 L-HOPE Program for Community-Based Total Learning Health Systems, Korea University, Seoul, Republic of Korea; 3 Department of Psychiatry, Seoul National University Bundang Hospital, Seongnam, Republic of Korea; 4 Department of Psychiatry, Seoul National University College of Medicine, Seoul, Republic of Korea; 5 Department of Psychiatry, College of Medicine, Soonchunhyang University Cheonan Hospital, Cheonan, Republic of Korea; 6 Department of Psychiatry, Soonchunhyang University College of Medicine, Cheonan, Republic of Korea; 7 Department of Psychiatry, Seoul Metropolitan Children’s Hospital, Seoul, Republic of Korea; 8 Department of Biological Sciences, Korea Advanced Institute of Science and Technology, Daejeon, Republic of Korea; 9 Center for Synaptic Brain Dysfunctions, Institute for Basic Science, Daejeon, Republic of Korea; 10 Laboratory of Genetics, University of Wisconsin-Madison, Madison, WI USA; 11 Department of Psychology, Korea University, Seoul, Republic of Korea; 12 Institute of Developmental and Regenerative Medicine, Department of Pediatrics, University of Oxford, Oxford, UK; 13 Department of Psychiatry and Behavioral Sciences, UCSF Weill Institute for Neurosciences, University of California, San Francisco, USA; 14 School of Biosystem and Biomedical Science, College of Health Science, Korea University, Seoul, Republic of Korea

**Keywords:** Familial phenotype deviation, De novo variants, Autism spectrum disorder, Neurodevelopment, Phenotypic variability

## Abstract

**Background:**

The phenotypic outcomes of de novo variants (DNVs) in autism spectrum disorder (ASD) exhibit wide variability. To date, no study has comprehensively estimated DNV effects accounting for familial phenotypic background.

**Methods:**

To evaluate DNV effects in a family-relative context, we defined within-family standardized deviations (WFSD) by subtracting phenotype scores of unaffected family members and standardizing the result. We applied this approach to 78,685 individuals from 21,735 families from ASD cohorts of diverse ancestries. We compared the distribution, associations with disruptive DNVs, and gene discovery results between WFSD and raw phenotype scores. We further performed outlier analysis based on WFSDs per gene to detect genes with high variability between families.

**Results:**

We observed that ASD probands with disruptive DNVs exhibited greater behavioral symptoms and lower adaptive functioning relative to their within-family unaffected members. Compared to raw phenotype scores, WFSD provided clearer associations with DNVs and enabled greater yield in DNV-enriched gene discovery, including 18 novel ASD-associated genes. Outlier analysis identified 11 genes with high intrafamilial variability in phenotypic effects, influenced by mutation sites within functional domains or exons.

**Conclusions:**

Familial DNV analysis provides accurate effect estimates, a reliable basis for predicting clinical outcomes, and precise support while minimizing confounding from family background. This approach improves the identification of ASD-associated genes with true phenotypic effects by reducing variability, as well as genes with genuine phenotypic heterogeneity across families driven by mutation site. These findings enhance our understanding of ASD phenotype variability and inform potential targets for intervention.

**Supplementary Information:**

The online version contains supplementary material available at 10.1186/s13073-025-01532-7.

## Background

Autism spectrum disorder (ASD) is a complex neurodevelopmental condition with a strong genetic basis [1]. Germline de novo variants (DNVs), identified in offspring but absent in parents, are significant contributors to ASD [2–4]. However, the effects of DNVs are highly variable, where the same recurrent mutation can lead to diverse phenotypic outcomes including intellectual disability (ID), heart defects, and facial dysmorphisms [5–7]. Recent advances in studies of DNVs in ASD and developmental delay (DD) have identified numerous genes [8–10] with substantial overlap between the diagnoses, such that 87% of ASD-associated genes are estimated to contribute to DD and 70% of DD-associated genes contribute to ASD [10, 11].

Familial background is a dominant modifier in the variable expressivity of genetic variants. In achondroplasia, where a gain-of-function missense mutation in *FGFR3* is the primary cause, height is consistently reduced but varies widely, and this variability correlates strongly with parental heights [12, 13]. Similarly, in ASD, neurodevelopmental profiles of probands with rare copy number variants correlate significantly with those of their parents [14–17]. To address these influences, studies compared phenotype scores of carriers to non-carrier family members to estimate variant effects. By accounting for shared genetic and environmental factors, this approach provides more accurate estimates of variant effects than deviations from population averages [14, 18–22]. However, most studies to date have not used within-family comparisons, limiting the precision of DNV effect assessments.

Even when familial background is considered, DNVs in the same gene can result in variable phenotypic outcomes owing to differences in the functional mechanism or effect size, for example, between protein domains or exons [23–27]. Gain-of-function *SCN2A* missense DNVs in the voltage-sensing domains are associated with infantile epileptic encephalopathy, while loss-of-function missense variants in the pore-forming domains and protein-truncating variants are associated with ID and/or ASD without infantile seizures [23]. Similarly, *SHANK3* protein-truncating variants in exons encoding synaptic scaffolding regions are associated with severe neurodevelopmental outcomes, whereas variants in other exons result in less severe symptoms [26, 27]. Incorporating familial background can help clarify the relative phenotypic variation associated with polygenicity or DNV genotype.

In this study, we adopted a family-based approach by investigating within-family standard deviation (WFSD), a normalized deviation of the proband’s phenotype score from their unaffected family members. Using data of 78,685 individuals in 21,735 families across diverse ancestries from the Korean Autism [28], Simons Simplex Collection (SSC) [29], and Simons Foundation Powering Autism Research for Knowledge (SPARK) [30] cohorts, we assessed WFSD of ASD core symptoms and adaptive abilities. By accounting for the polygenic effects of familial backgrounds, this approach could provide greater insights into the phenotypic impact of the specific DNV. Additionally, per-gene outlier analyses identified genes with high variability in intrafamilial deviations, likely reflecting genotype-specific functional differences. These findings provide a foundation for more accurate clinical interpretation of DNVs and suggest potential targets for precision intervention in ASD.

## Methods

### Cohorts

This study is based on whole-genome sequencing (WGS) and whole-exome sequencing (WES) data of 78,685 individuals from 21,735 full-trio ASD families across three cohorts: the Korean Autism (*n* = 2,605) [28], SSC (*n* = 9,082) [29], and *SPARK (*n = 67,098) [30] cohorts (Table 1). Among these, 24,050 were ASD probands, 11,165 were unaffected siblings, and 43,470 were parents.

**Table 1:** Sample information

Cohort	Data type	Participants, No				Families, No	ASD only, No. (%)^a^		
		ASD	Sibling	Parents	Total	Total	dnPTV carrier^b^	dnMIS carrier^c^	Non-carrier^d^
Korean	WES	61	67	122	250	61	2 (3.28)	4 (6.56)	55 (90.2)
	WGS	693	216	1346	2255	673	26 (3.75)	26 (3.75)	641 (92.5)
	WES + WGS	754	283	1468	2505	734	28 (3.71)	30 (3.98)	696 (92.3)
SPARK	WES	17,420	6757	30,534	54,711	15,267	606 (3.48)	457 (2.62)	16,357 (93.9)
	WGS	3496	2187	6704	12,387	3352	156 (4.46)	154 (4.41)	3186 (91.1)
	WES + WGS	20,916	8944	37,238	67,098	18,619	762 (3.64)	611 (2.92)	19,543 (93.4)
SSC	WGS	2380	1938	4764	9802	2382	141 (5.92)	110 (4.62)	2129 (89.5)
Total		24,050	11,165	43,470	78,685	21,735	931 (3.87)	751 (3.12)	22,368 (93.0)

Summary of sample characteristics of ASD probands, siblings, and parents from three sequenced cohorts (Korean, SPARK, SSC) using either whole-exome sequencing (WES), whole-genome sequencing (WGS), or both

^a^Number and percentage of ASD probands in each genetic category are presented as number (percentage)

^b^De novo protein-truncating variants (dnPTV) with LOEUF < 0.37

^c^De novo missense variants (dnMIS) with MPC ≥ 2

^d^Non-carriers: ASD probands without dnPTV or dnMIS

### Samples and genotyping

For the Korean Autism cohort, we collected DNA samples and clinical phenotype data from families with at least one child diagnosed with ASD by clinicians. The data collection took place across three major hospital sites in Korea: Seoul National University Bundang Hospital (SNUBH), which served as the primary center, along with Soon Chun Hyang University Hospital Bucheon (SCHBC) and Seoul Child Hospital (SCH). The ethics committees of SNUBH, SCHBC, and SCH IRB approved the study, with approval numbers as follows: SNUBH (B-1703–388–303 and B-2108–700–107), SCHBC (SCHBC 2018–04–020 and SCHBC 2022–04–016), and SCH (P01-201908-BM-02 and P01-202111–21–003). All phenotype data were cross-validated by clinical specialists. The collected data were anonymized and managed in accordance with the biorepository's standard operating procedures. The Korean cohort included 61 families (250 individuals) with WES data and 673 families (2255 individuals) with WGS data. For the SSC and SPARK cohorts, we downloaded genotype data and clinical data from SFARI Base (https://sfari.org/sfari-base). The SSC cohort included 2382 families (9082 individuals) with WGS data. The SPARK cohort comprised 15,267 families (54,711 individuals) with WES data and 3352 families (12,387 individuals) with WGS data. All procedures adhered to the ethical standards of the Helsinki Declaration, with informed consent obtained from all participants.

DNA was extracted from whole blood for the Korean and SSC cohorts and from saliva for the SPARK cohort. Sequencing was performed using Illumina platforms: HiSeq X for Korean WGS, NovaSeq 6000 for SPARK WES, and HiSeq X10 for SSC WGS. WES reads were aligned to the GRCh38 genome using BWA-mem, and variant calling was conducted with the Genome Analysis Toolkit (GATK) following best practices (v4.1.8.1 for Korean WES; v3.5 for SPARK and SSC), including variant quality score recalibration (VQSR). Korean WGS data were processed using the Illumina DRAGEN pipeline (v4.0.3). Joint genotyping was performed with the iterative gVCF genotyper for Korean WGS, GLnexus (v1.4.1) for SPARK, and GATK pipelines for SSC. Variants were filtered to include only those with “PASS” in the FILTER column and with quality metrics (GQ ≥ 20; DP ≥ 10 for SSC) to ensure high-quality calls. Additional filtering steps were applied to exclude low-complexity regions, split multiallelic sites, and remove large INDELs (allele length ≥ 50).

### DNV identification

DNVs were identified using the Hail 0.2 (https://hail.is/) de_novo() function on variants with an allele frequency (AF) below 0.01% in the gnomAD v3.1 non-neuro population, utilizing default parameters. For the Korean WGS cohort, we refined the DNV probability calculation by assessing variant origin and inheritance probabilities, adjusting the de novo probability threshold from 0.5 to 0.1 to align with GATK-processed DNV counts, referring to our previous work [28]. Quality filters were applied as follows: heterozygous SNPs required QUAL ≥ 7.5, GQmean ≥ 36, DPmean ≥ 34, and allele balance (AB) between 0.275 and 0.725; heterozygous indels required QUAL ≥ 10.51, gDP ≥ 3, and AB between 0.214 and 0.786. DNVs present in fewer than five individuals were retained. For the Korean WES data, only high/medium confidence DNVs were included with the following cutoffs:; heterozygous SNPs with Qual ≥ 135.58, GQmean ≥ 76, 0.77 ≥ AB ≥ 0.23, gDP ≥ 10; heterozygous indels with Qual ≥ 115.8, QD ≥ 6.64, MQ ≥ 32.15, SOR ≤ 2.35, gDP ≥ 13, 0.83 ≥ AB ≥ 0.17. DNVs present in fewer than two individuals were retained.

In the SSC WGS cohort, high/medium confidence DNVs were filtered with cutoffs based on prior studies [31]. We additionally filtered DNVs with internal allele count (AC) = 1, excluding outliers with DNV counts exceeding nine median standard deviations. The SPARK WGS and WES data were also filtered to retain high/medium confidence DNVs with AB < 0.8 and internal AC = 1, excluding outliers with DNV counts exceeding nine median standard deviations.

High-quality variants were annotated using Hail’s vep() function with Ensembl VEP version 109.3 and classified into protein-truncating variants (PTV), missense variants (MIS), and synonymous variants based on the most severe VEP consequence term. PTV included frameshift, splice acceptor/donor, and stop gain variants verified by the LOFTEE plugin, while MIS included missense and other protein-altering variants. The downstream analysis restricted PTVs in genes with loss-of-function observed over expected upper bound fraction (LOEUF) scores [32] < 0.37 and missense variants with missense badness, PolyPhen-2, constraint (MPC) [33] ≥ 2 to ensure functional significance.

### Polygenic score calculation

Following our previously described procedures [28], we derived high-quality common variants from WGS data using the following filters: genotype quality ≥ 20, depth ≥ 10, allele balance 0.2–0.8 for heterozygous and ≥ 0.95 for homozygous calls, call rate ≥ 95%, and Hardy–Weinberg equilibrium *P* ≥ 1 × 10^−6^. Variants with minor allele frequency > 0.05 among internal unrelated samples were retained. Polygenic scores (PS) were computed using PRScs [34] with the default parameters (global shrinkage phi = 1.0 × 10^−2^, gamma = (1,0.5)) and the HapMap3 SNP LD reference panel from European-ancestry UK Biobank samples. For ASD PS, we used GWAS summary statistics from Grove et al. [35], which included SSC and iPSYCH cohorts, and therefore computed ASD PS only in the Korean subset to avoid overlap-related inflation. For the educational attainment PS, we used European-ancestry GWAS summary statistics from Lee et al. [36] and computed scores in the entire cohort. Prior to SNP matching, WGS variants were lifted over to GRCh37 to harmonize genome build versions, and ambiguous SNPs as well as reverse-orientation INDELs were excluded. Polygenic transmission disequilibrium test (pTDT) was performed by comparing each proband’s polygenic score to the mid-parental mean PS. The deviation of proband PS from the parental mean was calculated, and the distribution of these deviations was tested against zero using one-sample t-tests. Differences in pTDT between de novo variant carrier groups were assessed using two-sample t-tests.

### Clinical measures

To evaluate phenotype distribution in ASD probands based on the presence of disruptive DNVs, we focused on two primary domains: ASD core symptoms and development-associated abilities (Table 2). ASD core symptoms were categorized into social communication deficits, restricted/repetitive behaviors, and total symptom severity (summed scores of social communication deficits and restricted/repetitive behaviors). Total symptom severity was assessed using the Autism Diagnostic Observation Schedule-2 [37, 38] (ADOS-2) calibrated severity scores (CSS) total scores, Social Responsiveness Scale [39] (SRS) T-scores, and Social Communication Questionnaire (SCQ) lifetime and current [40, 41] scores. Social communication deficits were measured with ADOS CSS Social Affect (SA) and Autism Diagnostic Interview-Revised [42] (ADI-R) domains A (social interaction) and B (communication), while restricted/repetitive behaviors were evaluated using ADOS CSS RRB, ADI-R RRB (domain C), and the Repetitive Behavior Scale—Revised [43] (RBSR). Development-associated functions included cognitive ability, assessed via full-scale IQ (FSIQ) [44, 45] and Leiter International Performance Scale-Revised IQ [46] (non-verbal IQ), adaptive ability measured by Vineland Adaptive Behavior Scales (VABS)-II [47] across five domains (total, communication, daily living, socialization, and motor skills), and motor coordination evaluated using the Development Coordination Disorder Questionnaire [48] (DCDQ).

**Table 2:** Phenotype distribution in probands

			Genetic subgroup, Mean (SD)^a^			ANOVA, *P*^b^
			dnPTV carrier	dnMIS carrier	Non-carrier	
ASD core symptom^c^	Total symptom severity	ADOS total	7.38 (1.67)	7.26 (1.65)	7.43 (1.71)	0.49
		SCQ current	14.92 (6.29)	16.52 (7.69)	16.26 (7.20)	0.18
		SCQ lifetime	20.79 (7.06)	20.76 (6.90)	20.84 (7.32)	0.96
		SRS	80.64 (10.0)	78.00 (10.6)	78.30 (11.1)	0.03
	Social communication deficits	ADOS SA	7.19 (1.81)	6.91 (1.78)	7.32 (1.77)	0.02
		ADIR A	19.87 (5.42)	20.47 (6.04)	20.24 (5.94)	0.65
		ADIR B verbal	16.13 (4.10)	16.12 (4.26)	15.98 (4.62)	0.89
	Restricted/repetitive behavior	ADOS RRB	7.36 (2.42)	7.81 (1.95)	7.39 (2.22)	0.08
		ADIR C	6.44 (2.68)	6.26 (2.24)	6.30 (2.57)	0.77
		RBSR	30.20 (18.8)	29.55 (17.9)	30.78 (19.0)	0.25
Development-associated^d^	Cognitive ability	FSIQ	74.11 (23.1)	74.27 (26.2)	81.34 (26.5)	1.41 × 10^−6^
		Non-verbal IQ	77.10 (23.0)	77.73 (25.7)	86.16 (25.8)	5.71 × 10^−9^
	Adaptive ability	VABS total	69.17 (14.7)	69.18 (16.1)	72.36 (14.9)	5.00 × 10^−8^
		VABS communication	69.95 (19.8)	69.72 (21.3)	73.20 (19.7)	1.90 × 10^−5^
		VABS daily living	71.86 (17.0)	71.32 (18.4)	75.30 (17.1)	3.24 × 10^−8^
		VABS socialization	66.70 (17.4)	66.89 (17.9)	69.14 (17.6)	1.00 × 10^−3^
		VABS motor skills	76.74 (14.9)	76.42 (15.5)	79.94 (15.4)	1.57 × 10^−4^
	Coordination	DCDQ	33.65 (10.6)	34.68 (11.9)	38.88 (12.5)	3.83 × 10^−7^

ASD probands were grouped into three genetic subgroups based on whether they carry de novo variants: dnPTV carriers (LOEUF < 0.37), dnMIS carriers (MPC ≥ 2), and non-carriers (no dnPTV or dnMIS). Statistical significance was assessed using one-way ANOVA across genetic subgroups

^a^Phenotype scores are presented as mean (standard deviation) unless otherwise indicated

^b^*P* values are from one-way ANOVA testing across the three genetic subgroups; *P* values < 0.05 are considered statistically significant

^c^ASD core symptoms include total symptom severity (ADOS, SCQ, SRS), social communication deficits (ADOS SA, ADI-R A & B), and restricted/repetitive behavior (ADOS RRB, ADI-R C, RBSR). Higher scores indicate greater symptom severity

^d^Development-associated phenotypes include cognitive ability (FSIQ, non-verbal IQ), adaptive ability (VABS), and coordination (DCDQ). Lower scores reflect greater functional impairment

We compared those 18 clinical phenotypes between ASD probands with de novo PTV (dnPTV), those with de novo MIS (dnMIS), and those without either dnPTV or dnMIS. We ran analysis of variance (ANOVA) tests and found that dnPTV or dnMIS carriers have significantly higher core symptom severity (SRS T) and lower developmental abilities (IQ, VABS, and DCDQ) (Table 2).

### Variable expressivity of DNVs

To examine expressivity of disruptive de novo variants (dnDIS), including dnPTV and dnMIS, we investigated the phenotypic profiles of ASD probands carrying dnDIS. We defined ID as FSIQ < 70, non-verbal IQ < 70 when FSIQ is not available, and VABS total < 70 when both FSIQ and non-verbal IQ are not available, and individuals with intellectual functioning higher than population averages as FSIQ ≥ 100, non-verbal IQ ≥ 100 when FSIQ is not available, and VABS total ≥ 100 when both FSIQ and non-verbal IQ are not available. We assessed the proportion of comorbid ID and higher-than-population ASD and the proportion of normative, mild to moderate, and severe symptoms using SRS norms (normative: SRS T < 60; mild to moderate: SRS T 60–75; severe: SRS T > 75) [39] in dnDIS carriers.

### Investigation of within-family standard deviation

To calculate WFSD, we selected clinical phenotypes assessed in both ASD probands and their unaffected family members across at least two cohorts. These phenotypes included SRS T-scores (available in the Korean and SSC cohorts), SCQ lifetime scores (available in the Korean, SSC, and SPARK cohorts), VABS scores (available in the Korean and SSC cohorts), ADOS CSS total, SA, and RRB sub-scores (available in the Korean cohort), and FSIQ (available in the Korean cohort). While both SRS T and SCQ lifetime scores measure overall symptom severity, SRS primarily reflects behavioral severity, whereas SCQ scores are more indicative of communicative severity. ADOS total scores provide an independent clinician-rated assessment of observed symptom severity, with the SA and RRB sub-scores capturing specific symptom domains including social impairment and repetitive behavior. The number of families included in each analysis varied: 2699 families for SRS, 7920 families for SCQ lifetime, 1952 families for VABS, 223 families for ADOS total, 215 families for ADOS SA, 215 families for ADOS RRB, and 127 families for FSIQ. Intrafamilial phenotype deviation was determined by comparing the proband’s scores with the mean scores of their unaffected family members and normalizing the differences by the SD of the general population (SD_SRS_ = 10, SD_VABS_ = 15, SD_FSIQ_ = 15). We applied the same normalization strategy across cohorts, given that previous studies have validated the use of K-SRS T-scores [49] and K-VBAS II scores [50]. For SCQ lifetime and ADOS scores, we used the SD derived from our dataset (SD_SCQ_ = 10.45, SD_ADOS_Total_ = 2.17, SD_ADOS_SA_ = 2.13, SD_ADOS_RRB_ = 2.61) due to the absence of established population norms.

Next, we compared WFSD of phenotype scores in ASD probands with dnPTV, with dnMIS, and those without either dnPTV or dnMIS using a generalized linear model (GLM) regression to evaluate differences in WFSD across carrier groups. When accounting for potential confounding factors, we conducted a GLM controlling for age, sex, and cohort to compare WFSD across genetic subgroups.

To comprehensively assess the neurodevelopmental impact of dnDIS, we constructed a two-dimensional (2D) space combining SRS and VABS WFSD scores. This framework enabled the simultaneous evaluation of symptom severity and adaptive abilities, offering a holistic view of neurodevelopmental profiles influenced by DNVs. With this framework, we estimated neurodevelopmental effects of total dnDIS and dnDIS in known gene sets [10] including ASD-associated genes, DD-associated genes, ASD-dominant genes (predominantly associated with ASD rather than DD), and DD-dominant genes (predominantly associated with DD rather than ASD). For the downstream WFSD outlier analysis, we utilized SRS T and VABS scores, which are normalized for age and sex.

To evaluate the distributional properties of raw SRS T-scores and intrafamilial deviations, we conducted normality tests using the Shapiro–Wilk, Anderson–Darling, and Kolmogorov–Smirnov methods. These tests assessed whether phenotype score distributions adhered to normality assumptions, which is essential for subsequent statistical analyses. The Shapiro–Wilk test statistic (W) ranges from 0 to 1, with higher values indicating greater adherence to normality. Conversely, higher Anderson–Darling (AD) and Kolmogorov–Smirnov (KS) test statistics reflect greater deviations from normality. *P*-values from these tests represent the probability of observing the data under the null hypothesis of normality; lower *p*-values (< 0.05) indicate non-normal distributions.

For association testing, we defined severe ASD phenotypes as those with raw SRS T-scores ≥ 76 (2.6 SD above the population mean) or equivalent intrafamilial deviations (≥ 2.6 WFSD). We compared the odds of carrying dnDIS between individuals with and without severe phenotypes using Fisher’s exact test to calculate odds ratios. Additionally, we identified genes enriched for dnDIS in cases meeting severe phenotype criterion. To further evaluate the functional relevance of these gene sets, we performed Gene Ontology (GO) enrichment analysis using the clusterProfiler R package (v4.12.6) [51]. Enrichment was assessed against the Biological Process categories in the GO database (downloaded July 2023), with multiple testing correction by the Benjamini–Hochberg method. Significantly enriched pathways were defined as those with adjusted *P* < 0.05. Enrichment results were compared between the raw score and WFSD definitions to identify pathways uniquely associated with each gene set. Finally, we conducted enrichment analyses for differentially expressed genes in the developing human brain, encompassing 39 clusters across nine cell types: radial glia, neuroblasts, excitatory neurons, inhibitory neurons, astrocytes, microglia, oligodendrocytes, oligodendrocyte precursor cells, and endothelial cells [52].

### Phenotype outcome outlier analysis

To identify genes with highly variable impacts on phenotypes between unrelated families, we performed outlier analyses for each variant type (dnMIS and dnPTV) across raw SRS T and VABS scores and SRS and VABS WFSD, resulting in eight distinct analyses. For each combination, we calculated the median absolute deviation (MAD) of the phenotype outcomes among all variant carriers within each gene. Genes were classified as outliers if they met the following criteria:

1. MAD exceeded the overall mean MAD by more than two SDs for the specific variant type and phenotype.
2. Variants within the gene were observed in more than two samples.
3. (For WFSD only) WFSD observations were above the 95th percentile for the respective phenotype and variant type.

Identified outlier genes were further analyzed for the location of mutations within specific functional domains or exons using the UCSC Genome Browser (https://genome.ucsc.edu/). By conducting analyses separately for each variant type and phenotype, we ensured a comprehensive assessment of gene-specific impacts on neurodevelopmental profiles.

## Results

### Family-based analyses explain varying phenotype outcomes of a de novo mutation

We analyzed WGS/WES and phenotype data of 78,685 individuals from 21,735 families with ASD probands across diverse ancestries from the Korean, SSC, and SPARK cohorts (Table 1). The distributions of exonic DNVs per individual were comparable between WES and WGS datasets in all cohorts (Additional file 1: Fig. S1). Among the 24,050 ASD probands, approximately 10% carried dnDIS, including damaging dnPTV (LOEUF [32] < 0.37) and dnMIS (MPC [33] ≥ 2) (Table 1). Probands carrying dnDIS demonstrated more severe clinical profiles than non-carriers with ASD, including significantly lower developmental abilities across cognitive, adaptive, and motor coordination domains (Table 2). Although most ASD core symptom measures, especially social communication deficits, showed a trend toward attenuated severity in probands carrying dnDIS compared to non-carriers, consistent with previous findings [53], SRS T-scores were significantly higher in dnDIS carriers (Table 2).

**Fig. 1:**
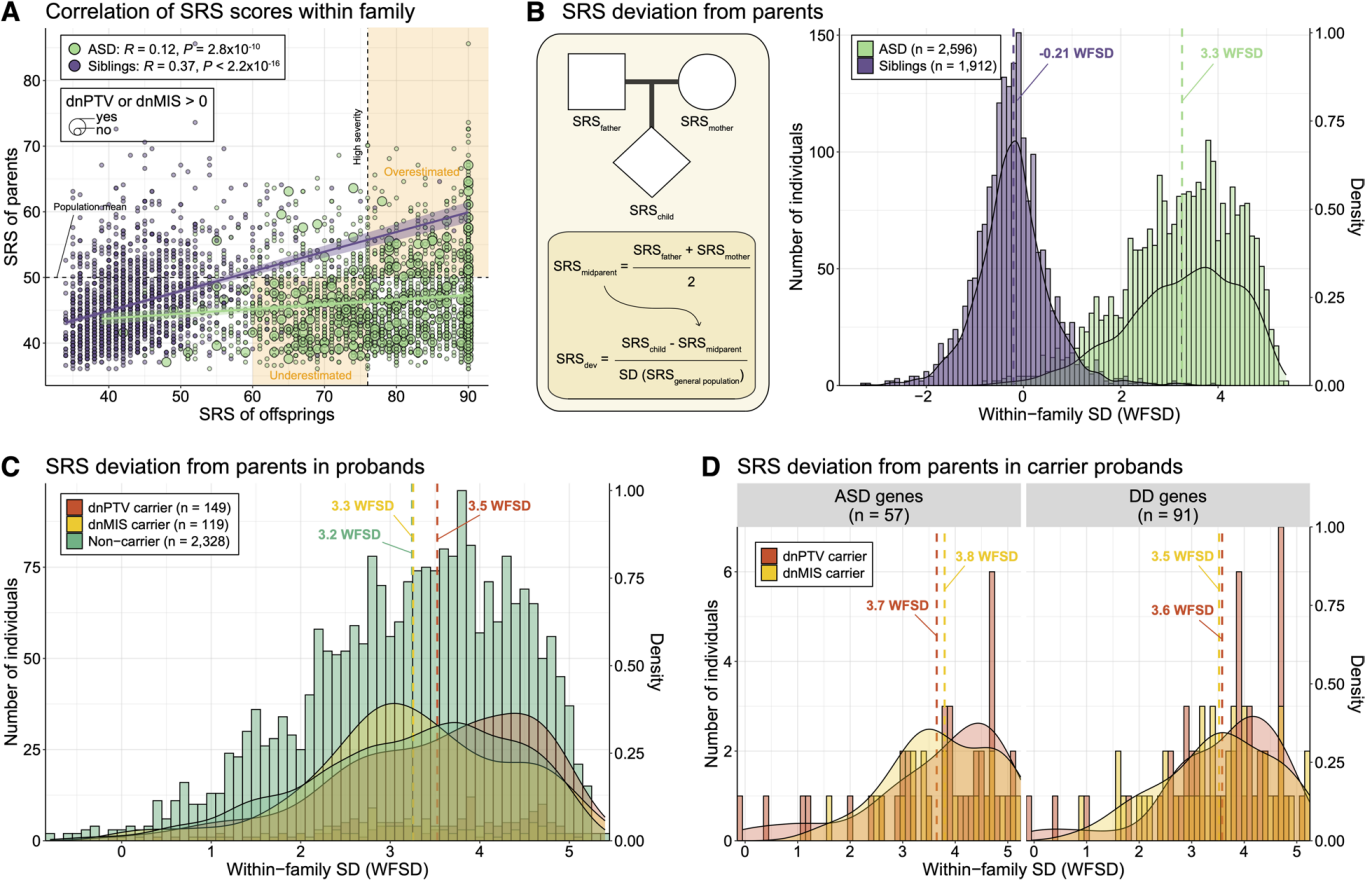
Intrafamilial deviation of social responsive scale from parents. **A** Correlation of social responsiveness scale (SRS) T-scores between offspring and parents within families. The effect of de novo variants (DNVs) may be underestimated when the offspring exhibit milder SRS severity but their parents have lower-than-average SRS. Conversely, effects may be overestimated when the offspring have high SRS severity and their parents also have high SRS T-scores. **B** Calculation of within-family standard deviation (WFSD) for SRS in offspring from parents. WFSD is calculated by subtracting the mean parental SRS T-score from the offspring’s score and dividing by the general population SD. The histogram shows the distribution of SRS deviations in ASD probands (*n* = 2598) and unaffected siblings (*n* = 1910). **C** SRS deviation distributions among genetic subgroups in ASD cases, showing greater deviations in carriers of de novo protein-truncating variant (dnPTV) and missense (dnMIS). **D** SRS deviations stratified by gene category: ASD-/DD-associated genes

However, there was substantial variability in the clinical outcomes of dnDIS carriers. For instance, 45.5% of dnDIS carriers had comorbid ID, while 8.3% had intellectual functioning higher than population averages, and 66.6% exhibited severe symptoms, while 33.4% exhibited normative to moderate symptoms. To investigate the modifying effects of familial background on this variable expressivity, we examined correlations between mid-parental mean and offspring SRS T-scores across all fully phenotyped families (*N* = 2699). Consistent with previous findings [14, 54], significant correlations were observed in both ASD cases (*R* = 0.12, *P* = 2.8 × 10^−10^) and unaffected siblings (*R* = 0.37, *P* < 2.2 × 10^−16^) (Fig. 1A). Among the 26% of dnPTV and dnMIS carriers who exhibited normative to moderate SRS severity (SRS T 60–75), 87% of their parents scored lower-than-average on SRS (SRS T < 60) [39]. Without accounting for familial baselines, such cases may lead to underestimation of DNV effects.

To address this, we calculated WFSD by subtracting the mid-parental mean SRS T-score from the proband’s score and normalizing it by the general population SD (SD = 10) [39] (Fig. 1B). ASD probands demonstrated a mean increase in SRS deviation of 3.3 WFSD, a significant shift relative to their parents. In contrast, unaffected siblings showed a small negative deviation of − 0.21 WFSD. We next investigated SRS deviation based on whether probands carry dnPTV, dnMIS, or none. Among the subgroups, dnPTV carriers had the largest deviation from their parents (3.5 WFSD), followed by dnMIS carriers (3.3 WFSD) and non-carriers (3.2 WFSD) (Fig. 1C; Additional file 2: Table S1). We further stratified SRS deviations based on the gene categories harboring dnPTV or dnMIS, using previously characterized ASD-associated genes [10] (*N* = 72) and DD genes [9] (*N* = 285). Greater deviations were observed for ASD-associated genes compared to DD genes for both dnPTV carriers (3.7 vs. 3.6 WFSD) and dnMIS carriers (3.8 vs. 3.5 WFSD), although the differences were not significant (Fig. 1D; Additional file 1: Fig. S2A; Additional file 2: Table S1). We also examined whether the number of dnPTV or dnMIS variants per individual was associated with increased SRS deviation to test for potential cumulative effects. However, no clear trend in deviation was observed between those with one versus more than one variant, likely due to the extremely small number of such multi-hit carriers (e.g., *n* = 2 for dnPTV > 1). To further explore the relationship between DNV burden and inherited liability, we calculated pTDT scores based on ASD polygenic scores [35]. Non-carrier probands showed significantly elevated pTDT compared to 0 (*P* < 0.001, one-sample t-test), suggesting over-transmission of common variant risk. In contrast, DNV carriers did not show significant over-transmission. Additionally, we found that dnPTV carriers with mutations in ASD-associated genes exhibited significantly lower pTDT compared to non-carriers (*P* = 0.02, two-sample *t*-test), consistent with a liability threshold model (Additional file 1: Fig. S3). This result supports a polygenic architecture of ASD, where rare and common variants collectively influence phenotypic severity.

**Fig. 2:**
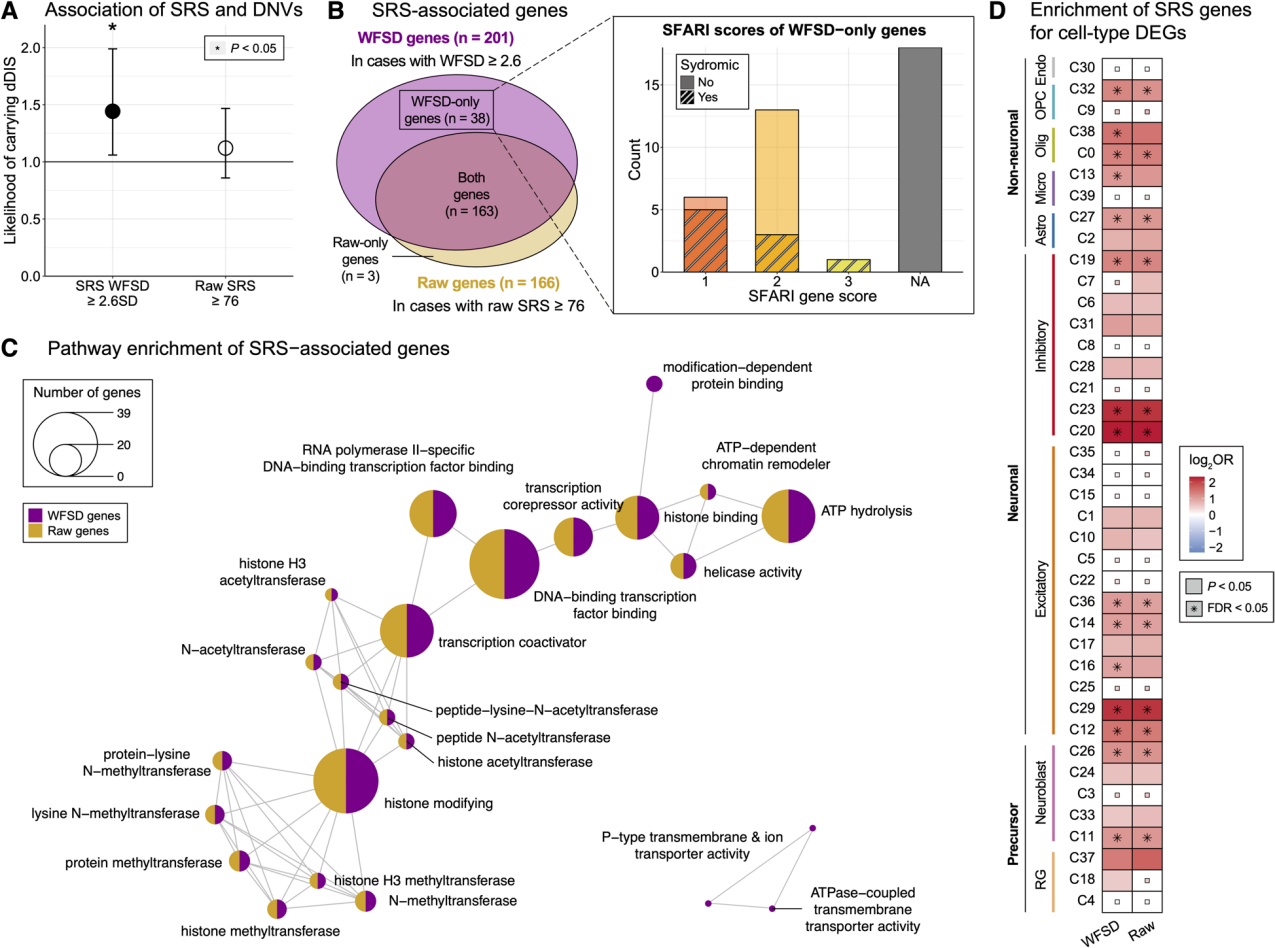
Comparison of gene associations using WFSD and raw phenotype scores. **A** Enrichment for disruptive de novo variants (dnDIS) carriers with social responsiveness scale (SRS) within-family standard deviation (WFSD) ≥ 2.6SD and raw SRS T-scores ≥ 76. **B** Number of genes enriched for dnDIS in ASD cases using each definition. Color represents group origin (purple: WFSD, yellow: raw, both: overlapping). Inset shows SFARI gene rankings for genes uniquely identified with WFSD ≥ 2.6SD. **C** Pathway enrichment analysis of SRS-associated genes using GO molecular function terms. Circle size indicates the number of associated genes. **D** Enrichment of SRS-associated genes in cell type–specific differentially expressed gene sets from the developing human brain. Color scale indicates log odds ratio of overlap; asterisks denote FDR < 0.05

Compared to raw SRS T-scores, WFSD distribution was closer to normality with fewer outliers across genetic subgroups in probands (Additional file 1: Fig. S4). To further evaluate the utility of WFSD, we assessed the associations between dnDIS and severe SRS profiles using two definitions: raw SRS T-scores ≥ 76 (2.6 SD above the population mean) and intrafamilial deviations ≥ 2.6 WFSD. Stronger associations were observed with WFSD (OR = 1.39, 95% CI = 1.02–1.91) than raw T-scores (OR = 1.13, 95% CI = 0.86–1.50) (Fig. 2A). We also investigated genes that have dnDIS in ASD probands with severe SRS profiles using both definitions. More dnDIS-enriched genes were identified using the WFSD definition (*N* = 201) than the raw T-score definition (*N* = 167), with 38 genes uniquely identified in the WFSD group (Fig. 2B; Additional file 3: Table S2). Of these, 20 were previously associated with ASD in the SFARI gene database (https://gene.sfari.org) [55], and 18 were novel.

To assess the functional relevance of SRS-associated genes, we examined pathway enrichment for genes identified under each definition (Fig. 2C). Many pathways were shared across both groups, including those related to transcriptional regulation, chromatin remodeling, and histone modification. These are well-established ASD-associated pathways [10]. Among the pathways uniquely enriched in WFSD-only genes, modification-dependent protein binding involves recognition of post-translationally modified proteins. This pathway included several chromatin-related regulators uniquely found in the WFSD-defined severe group, including *CBX4*, *KMT2E*, *ZMYND11*, and *PRPF8*. *CBX4* is a component of the Polycomb Repressive Complex 1, which is associated with NDD and has previously been highlighted as an ASD risk gene in network-based analyses [56, 57]. *ZMYND11* and *KMT2E* have been implicated in ASD and NDD through large-scale mutation studies and clinical case reports [58, 59]. While *PRPF8* has not been directly linked to ASD in prior genetic studies, it has been identified as an interactor of the high-confidence ASD gene *STXBP1* in a protein–protein interaction network [60]. Another uniquely enriched pathway, P-type/ATPase-coupled transmembrane transporter activity, included *ATP1A1* and *ATP2B2*, both uniquely identified in the WFSD-defined group. This pathway has also been implicated in schizophrenia through enrichment of ATPase-related functions among genes carrying loss-of-function variants [61]. Both *ATP1A1* and *ATP2B2* have been previously linked to neurodevelopmental disorders, including phenotypes such as intellectual disability, epilepsy, and ASD [62, 63].

Given the bimodal distribution of WFSD, we further compared individuals in the better-than-expected subgroup (SRS WFSD < 2.0) and the severe subgroup (SRS WFSD ≥ 2.6). The severe subgroup tended to have a higher burden of disruptive DNVs than the better-than-expected subgroup, and genes carrying such variants showed enrichment for both WFSD-only and well-established ASD-associated pathways (Additional file 1: Fig. S5). In contrast, the better-than-expected subgroup had a significantly higher frequency of less deleterious missense variants (MPC < 1) than the severe subgroup. In addition, genes carrying disruptive DNVs in this subset had no clear convergence in functional profiles, indicating more heterogeneous effects (Additional file 1: Fig. S5). When comparing dnDIS-enriched genes with cell type-specific differentially expressed genes (DEGs) in the developing human brain [52], we observed similar enrichment patterns across both raw and WFSD definitions (Fig. 2D; Additional file 3: Table S2). However, significant enrichments (FDR < 0.05) in microglia and oligodendrocyte clusters were exclusively observed in the WFSD-defined severe SRS group.

### Estimating the effect size of de novo variants on neurodevelopmental profiles

We further examined SRS deviations in probands from unaffected siblings within the same family (Additional file 1: Fig. S6A). Similar to deviations calculated from parents, dnPTV carriers exhibited the largest deviation of 3.9 WFSD, while dnMIS carriers and non-carriers showed deviations of 3.5 WFSD. For dnPTV carriers, the deviation increased to 4.0 WFSD when restricted to ASD/DD-associated genes (Additional file 1: Fig. S6B). For dnMIS carriers, deviations reached 3.9 WFSD in ASD genes and 3.7 WFSD in DD genes. Although deviations from siblings were marginally greater than those from parents (Additional file 1: Fig. S7; Additional file 2: Table S1), the patterns were nearly identical. These results were consistent across the proband’s genetic subgroup within the family, further validating the use of unaffected siblings as reliable proxies when parental phenotype data are unavailable.

We evaluated deviations in other clinical phenotypes assessed in unaffected siblings. Specifically, we analyzed VABS total, SCQ lifetime, ADOS CSS total, SA, RRB, and FSIQ scores, calculated following the same procedure as for SRS. Mean VABS scores for ASD probands were negatively shifted to the left relative to their siblings by 2.1 WFSD for dnPTV carriers, 2.2 WFSD for dnMIS carriers, and 2.0 WFSD for non-carriers (Additional file 1: Fig. S8A; Additional file 2: Table S1). Similarly, SCQ lifetime scores for probands were shifted to the right relative to siblings by 1.8 WFSD for both dnPTV and dnMIS carriers and 1.7 WFSD for non-carriers (Additional file 1: Fig. S8C; Additional file 2: Table S1). This pattern was more evident for ASD/DD-associated genes (Additional file 1: Fig. S8B, D). Although this pattern was more attenuated in ADOS and FSIQ WFSD due to the limited sample size, similar trends were observed, with ADOS scores shifted to the right and FSIQ scores shifted to the left relative to those of siblings (Additional file 1: Fig. S8E–L). The smaller number of observations primarily reflects practical challenges in obtaining these assessments, such as limited cooperation among participants and variability in clinical protocols across sites.

**Fig. 3:**
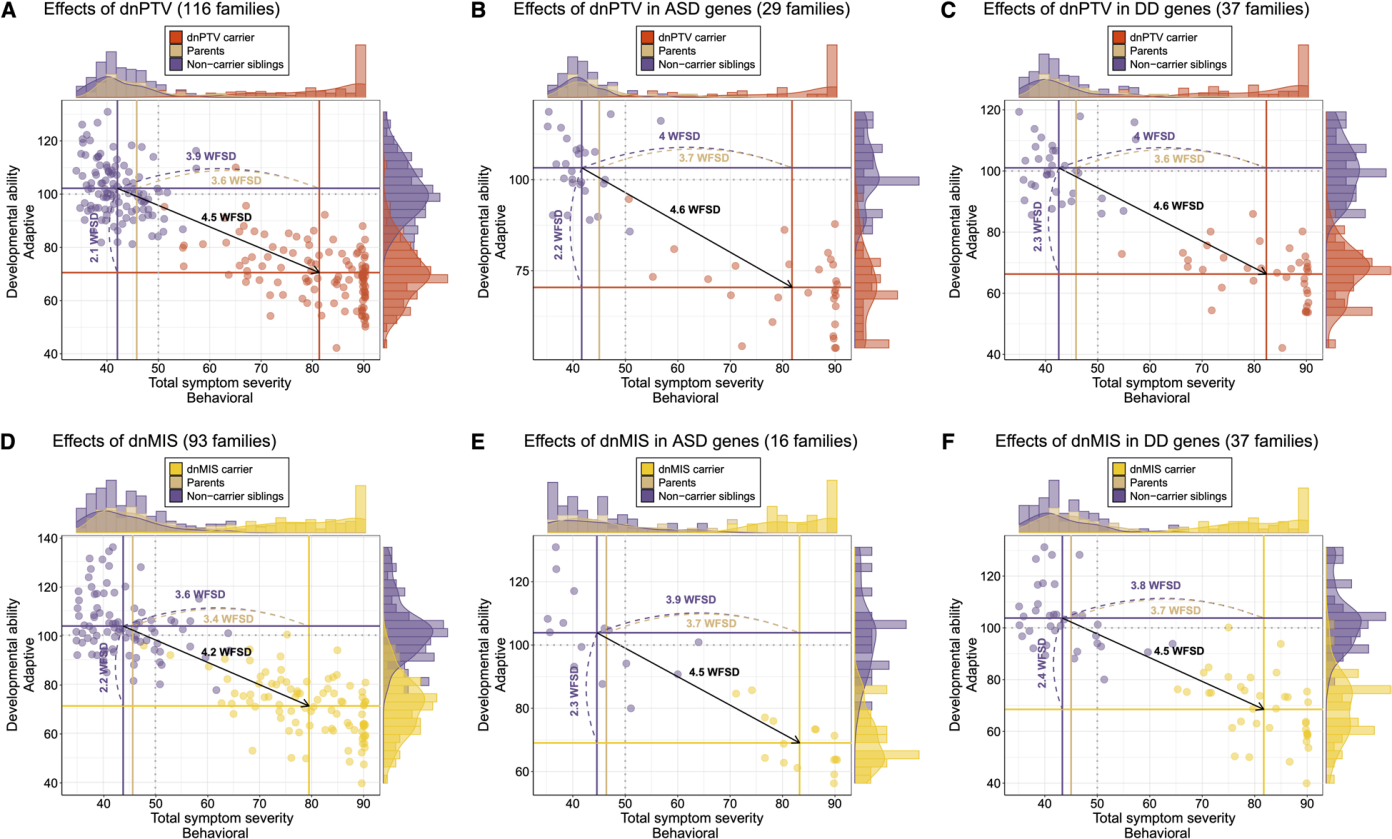
Effects of de novo variants on neurodevelopmental profiles. **A** Neurodevelopmental profile deviation (behavioral severity vs adaptive ability) for ASD probands with de novo protein-truncating variant (dnPTV) (*n* = 116 families) in a 2D space defined by behavioral symptom severity and adaptive ability (behavioral symptom severity: social responsiveness scale; adaptive ability: Vineland adaptive behavior scale), showing a shift of 4.5 within-family standard deviation (WFSD) towards higher behavioral severity and lower adaptive ability. **B** Profile deviation in ASD probands with dnPTV located in ASD-associated genes (*n* = 29 families), showing a shift of 4.6 WFSD. **C** Profile deviation in ASD probands with dnPTV in developmental disorder (DD)-associated genes (*n* = 37 families), showing a shift of 4.6 WFSD. **D** Profile deviation in ASD probands with de novo missense (dnMIS) (*n* = 94 families), showing a shift of 4.2 WFSD. **E** Profile deviation in ASD probands with dnMIS located in ASD-associated genes (*n* = 16 families), showing a shift of 4.5 WFSD. **F** Profile deviation in ASD probands with dnMIS located in DD-associated genes (*n* = 37 families), showing a shift of 4.5 WFSD

To comprehensively estimate the effect size of dnDIS on neurodevelopmental profiles, we constructed 2D space combining SRS T and VABS scores. The magnitude and direction of proband deviations were measured relative to unaffected siblings and parents within families. In the 2D space, ASD probands with dnPTV exhibited a diagonal shift of 4.5 WFSD, representing simultaneous increases in behavioral symptom and decreases in adaptive ability (Fig. 3A). This deviation increased slightly to 4.6 WFSD for dnPTV in ASD/DD-associated genes (Fig. 3B, C). In contrast, dnMIS carriers showed smaller overall deviations, with shifts of 4.2 WFSD across all genes and 4.5 WFSD for ASD/DD-associated genes (Fig. 3D–F). The largest neurodevelopmental impacts for both dnPTV and dnMIS carriers were observed when the variants were in DD genes predominantly associated with DD compared to ASD [10] (Additional file 1: Fig. S9).

### Genes with variable intrafamilial impacts

**Fig. 4:**
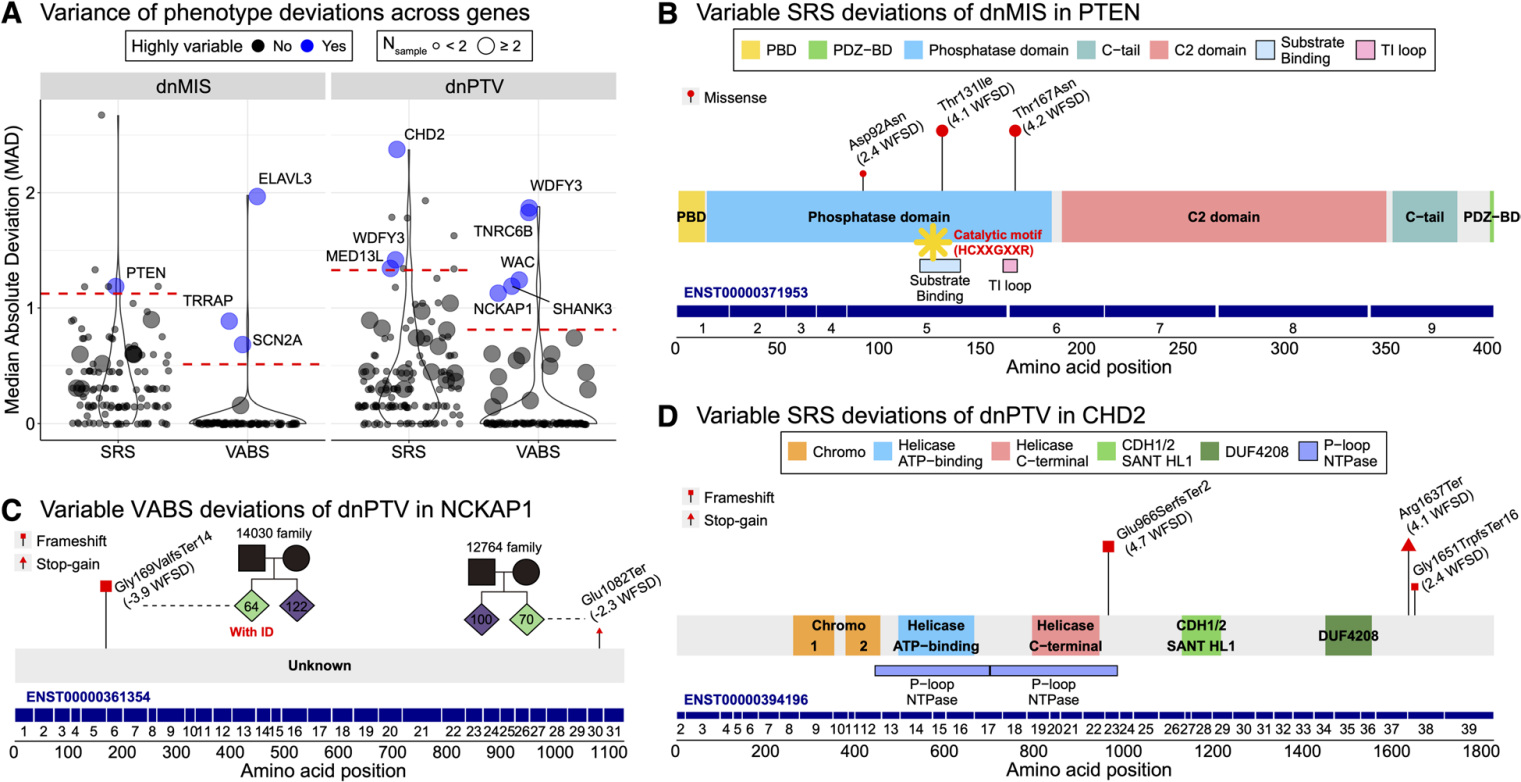
Genes with variable expressivity between families for neurodevelopmental profiles. **A** Median absolute deviation (MAD) of phenotype outcomes of de novo missense (dnMIS) and protein-truncating variant (dnPTV) for each gene, highlighting outlier genes with high variability in intrafamilial impacts. **B** Variable social responsiveness scale (SRS) deviation outcomes in unrelated ASD probands carrying dnMIS in different functional domains of *PTEN*. Functional domains and exons are indicated, demonstrating how mutation position influences phenotypic outcomes. Red points represent amino acid positions of missense variants, and the size of point and length of stem correspond to the effect size of phenotype deviations. **C** Variable Vineland adaptive behavior scale (VABS) deviation outcomes in unrelated ASD cases carrying dnPTV in different functional domains of *NCKAP1*. The pedigree illustrates how the impact of variants may be underestimated when examining raw VABS scores alone. Red rectangles indicate frameshift variants, and red triangles indicate stop-gain variants. **D** Variable SRS deviation outcomes in unrelated ASD cases carrying dnPTV in different functional domains of *CHD2*

To identify genes with highly variable impacts, we conducted outlier analysis for dnMIS and dnPTV using SRS and VABS. Among 1328 tested genes, 11 genes had variable intrafamilial impacts between unrelated families (Fig. 4A; Additional file 4: Table S3). For dnMIS variants, four genes exhibited high variability in intrafamilial deviations: *PTEN* for SRS; *SCN2A, ELAVL3*, and *TRRAP* for VABS. Among genes with dnPTV, seven genes showed high variability: *MED13L, WDFY3,* and *CHD2* for SRS; *SHANK3, NCKAP1, WAC, TNRC6B*, and *WDFY3* for VABS.

Among the genes with dnMIS and variable SRS WFSD between families, *PTEN* stood out as a notable example (Fig. 4B). *PTEN* is associated with a range of clinical outcomes, including ASD and cancers [64, 65]. Missense variants within its phosphatase domain, particularly those affecting the substrate-binding and TI loop regions, exhibited significantly more severe SRS deviations (p.Thr131Ile: 4.1 WFSD; p.Thr167Asn: 4.2 WFSD) than other *PTEN* dnMIS variants (p.Asp92Asn: 2.4 WFSD). Consistent with these observations, prior multi-model functional assays demonstrated that p.Thr131Ile exhibits near-complete loss-of-function across diverse cellular models, reflecting severe catalytic disruption, while p.Thr167Asn causes substantial impairment of phosphatase activity and partial protein instability [66]. In contrast, p.Asp92Asn retained partial catalytic activity in yeast reconstitution assays [67], consistent with the more modest phenotypic impact we observed. These findings align with previous reports demonstrating that the TI loop and adjacent catalytic motifs in the substrate-binding region are essential for determining the substrate specificity and enzymatic activity of *PTEN*, which in turn regulate critical downstream signaling pathways [68, 69]. Disruption of these regions likely amplifies the neurodevelopmental impact of *PTEN* variants, contributing to the observed phenotypic variability in ASD probands. Similarly, the other three dnMIS outlier genes also exhibit clinical variability depending on mutations in different functional domains [23, 70, 71].

For dnPTV variants, *NCKAP1* displayed substantial variability in its impact on VABS intrafamilial deviations (Fig. 4C). Two PTVs, p.Gly169ValfsTer14 and p.Glu1082Ter, exhibited markedly different VABS deviations, at − 3.9 WFSD and − 2.3 WFSD, respectively. These variants occurred in exon 5 and exon 30 out of total 31 exons. While both exons have similar transcript inclusion, the frameshift in the earlier exon likely caused greater disruption, potentially destabilizing downstream exons, and most transcripts, leading to greater phenotypic severity. This case is particularly noteworthy because, when examining raw VABS scores, the variability between these unrelated cases appears minimal (64 vs. 70), making it easy to overlook. Recent studies link *NCKAP1* PTV and MIS to varying severities of ASD [72], but its functional domains remain poorly understood, warranting further investigation into the mechanisms driving this variability.

Another example of highly variable dnPTV impacts was observed in *CHD2*, influencing SRS intrafamilial deviations (Fig. 4D). Recent studies indicate that while reported mutations in *CHD2* do not cluster within its known functional domains, the C-terminus, which enhances DNA binding and stimulates ATPase activity, is enriched with mutations [73, 74]. All three dnPTV were in the C-terminus and exhibited SRS deviations ranging from 2.4 WFSD to 4.7 WFSD. Among these, p.Glu966SerfsTer2 frameshift occurring in the earliest exon regulating the P-loop ATPase region had the most severe outcome (4.7 WFSD). In contrast, two mutations in the penultimate exon—a stop-gain (p.Arg1637Ter) and a frameshift (p.Gly1651TrpfsTer16)—resulted in deviations of 4.1 WFSD and 2.4 WFSD, respectively. The milder effect of the latter may reflect partial preservation of protein function, as frameshift mutations affecting less than 25% of the coding sequence can escape nonsense-mediated decay [75]. The remaining five dnPTV outlier genes have also been reported to show variable expressivity in prior literature, further validating our framework [26, 27, 76–79].

## Discussion

We measured the effect sizes of DNVs on behavioral symptom severity and adaptive functioning considering familial background leveraging large and ancestrally diverse ASD cohorts (Additional file 1: Fig. S10). This approach improved genotype–phenotype associations and led to the discovery of 18 novel ASD-associated genes. Furthermore, we identified 11 genes with high variability in intrafamilial effects, underscoring the importance of specific mutation sites in shaping the phenotypic heterogeneity in ASD.

The use of WFSD provided less variable and more robust associations with ASD than raw phenotype scores, indicating improved biological interpretability (Additional file 1: Fig. S4, Fig. 2). Comparison of gene-level effects of dnPTVs on SRS WFSD with external gene annotations (SFARI Gene) and effect size estimates [80] demonstrated largely consistent enrichment patterns, supporting the validity of our approach (Additional file 1: Fig. S11). Notably, among genes in the 10th decile of WFSD for dnPTV carriers, *SPAG9*, *UNK*, and *TNFRSF8* were neither listed in the SFARI Gene database [55] nor included in the gene-level effect size estimates by Rolland et al. [80]. These genes have primarily been implicated in cancer but were also reported in ASD probands or shown to regulate neurogenesis [81–83]. Additionally, our findings support the reliability of using unaffected siblings as proxies for parental data when calculating WFSD, especially when parental phenotypic information is unavailable.

Although utilizing WFSD showed a reduced variance of DNV effects on a gene-level as compared to raw phenotype scores (Additional file 1: Fig. S12; Additional file 4: Table S3), variability between families persisted due to differences in functional domains and exon positions. Outlier analysis of intrafamilial deviations helped identify such genes, suggesting candidates for future studies. When we broadened the analysis to include less- or non-damaging dnMIS (MPC < 2) and dnPTV (LOEUF ≥ 0.37), dnMIS exhibited greater variability than dnPTV as expected, likely due to their regional specificity (Additional file 1: Fig. S13; Additional file 4: Table S3). Furthermore, for missense variants, this variability often reflected domain-specific effects consistent with pathogenic hotspots or critical functional motifs. For protein-truncating variants, positional effects within the transcript, including the likelihood of nonsense-mediated decay escape, likely contributed to differential outcomes across families. This observation is consistent with previous work demonstrating that the exon-level position of mutations can be a major predictor of phenotypic similarity [84]. These mechanisms, together with isoform-specific expression and splicing, may underlie some of the observed heterogeneity, although larger sample sizes will be needed to systematically assess their impact.

However, our study has several limitations. SRS data were unavailable for the SPARK, which may reduce cohort diversity. Moreover, there were negligible contributions of sex, age, and the location of assessment for the variance in WFSD. For example, SRS deviations were larger in females with ASD, school-age probands, and the SSC cohort (Additional file 1: Fig. S14) and VABS deviations were larger in females with ASD, school-age probands, and the Korean cohort (Additional file 1: Fig. S15). However, the adjustment for these covariates did not change the relative WFSD differences between dnPTV, dnMIS, and non-carriers (Additional file 1: Fig. S16). Parental SRS T-scores were lower than the general population mean, and sibling VABS scores were higher than the general population mean, suggesting that comparisons with general population norms might underestimate the DNV effects. However, we cannot exclude the possibility of ascertainment bias being introduced, as participating families could have higher education levels and lower social impairments than the broader population. To evaluate whether such bias could systematically influence the estimated DNV effects, we conducted exploratory analyses testing interactions between parental or sibling phenotypes and DNV carrier status, which did not show any significant interaction effects (Additional file 1: Fig. S17A, C). Additionally, mean parental educational attainment polygenic scores were not correlated with within-family deviations (Additional file 1: Fig. S17B, D). These results suggest that while ascertainment bias remains a potential limitation, it did not have a detectable impact on the estimation of DNV effects in this study. Additionally, while we focused on two key phenotypic domains—SRS and VABS, which are normalized across age and sex—broadening the scope to a wider range of neurodevelopmental phenotypes could provide a comprehensive understanding of ASD profiles.

Our findings have significant clinical and biological implications. By providing accurate estimates of the effects of dnPTV and dnMIS, our approach can inform interventions, predictions, and treatment strategies tailored to individual genetic profiles. The identification of candidate genes associated with diverse clinical outcomes enhances our understanding of ASD etiology. Some genes with significant impacts were missed when using raw scores alone, underscoring the value of incorporating intrafamilial deviations in genetic analyses. Notably, 18 genes not listed in the SFARI Gene Data Base [55] were recently reported to be associated with neurodevelopmental disorders [85–91], emphasizing the need for functional studies.

We suggest that future studies integrate intrafamilial deviations to account for familial background effects. WFSD may facilitate personalized variant interpretation or support genetic counseling frameworks. Investigations into the sources of familial influence, such as inherited variants, environmental factors, or their interplay, would deepen our understanding. Additionally, while WFSD provides more specific estimates of DNV effects, the substantially smaller subset of probands with complete two-generation or sibling phenotyping imposes a further limitation. For example, while SRS scores were available in WFSD for a comparable number (Raw *N* = 2907; WFSD *N* = 2715), VABS data were not (Raw *N* = 10,232; WFSD N = 1955). This discrepancy likely contributed to missing signals in deviation-based analyses. Among the 28 genes that showed variable impacts on VABS raw scores but were un-assessable by WFSD (Additional file 1: Fig. S12), *PTEN* represents an informative example. In this case, individuals harboring mutations at residues Arg15 and Ile101 showed markedly low raw VABS scores (23 and 27, respectively). The Arg15 variant disrupts the PIP3-binding domain that mediates membrane localization and substrate interaction, while Ile101 impairs folding of the phosphatase domain and reduces catalytic activity. Both missense mutations have been shown in functional studies to cause severe loss of function [66]. These findings support the possibility that some true signals were undetected due to incomplete family phenotyping. Collecting detailed family-based phenotypic assessments, including standardized measures such as SRS and VABS from parents and siblings, alongside functional studies of variant effects, will be essential to validate and extend these observations. Addressing these questions will advance our understanding of the neurobiological mechanisms underlying ASD variability and contribute to the development of precise support and interventions.

## Conclusions

Accounting for familial background enables clinicians to predict the phenotypic outcomes of specific DNVs. This approach provides valuable insights into the biological mechanisms underlying ASD, enabling precise support and effective intervention strategies for individuals and families affected by ASD.

## Supplementary Information

Additional file 1: 17 supporting Figures S1-S17. Captions for each Fig. S1 to S17 are given within the file.

Additional file 2: Table S1. This table contains WFSD of each genetic subgroup: SRS deviations from parents (Table S1A), SRS deviations from siblings (Table S1B), VABS deviations from siblings (Table S1C), SCQ lifetime deviations from siblings (Table S1D), ADOS CSS total deviations from siblings (Table S1E), ADOS CSS SA deviations from siblings (Table S1F), ADOS CSS RRB deviations from siblings (Table S1G), and FSIQ deviations from siblings (Table S1H).

Additional file 3: Table S2. This table contains enrichment results of dnDIS in ASD probands with severe SRS profiles (Table S2A), lists genes enriched in these probands (Table S2B), and shows enrichment results of each SRS gene set with GO pathways (Table S2C) and with cell-type specific DEGs in the developing human brain (Table S2D).

Additional file 4: Table S3. This table contains MADs of phenotype outcomes of DNVs per gene: SRS deviations (Table S3A), and raw SRS scores (Table S3B).

## Data Availability

Due to local privacy laws and privileged human information, all requests for the raw genomic data from the Korean Autism cohort are subject to prior approval from the Institutional Review Board at Seoul National University Bundang Hospital. Interested researchers should submit a request to Dr. Hee Jeong Yoo, along with a detailed research plan outlining proposed analyses and data anonymization procedures. This plan will be reviewed by the IRB and data sharing committee at Seoul National University Bundang Hospital, with approval typically granted within two months. Upon approval, the requester will be added to the IRB as a collaborator for secure data sharing. Genetic and phenotypic data for the SSC and SPARK cohorts can be accessed by applying at https://base.sfari.org [92]. All major analysis scripts and code used to generate key figures will be made publicly available via Zenodo (10.5281/zenodo.15838146) [93]. Additionally, summary-level statistics from the WFSD analyses and curated gene lists used in this study will be provided as Supplementary Tables to ensure reproducibility and transparency.

## Abbreviations

DNV: De novo variant
ASD: Autism spectrum disorder
ID: Intellectual disability
DD: Developmental delay
WFSD: Within-family standard deviation
dnPTV: De novo protein-truncating variants
dnMIS: De novo missense
SRS: Social Responsiveness Scale
VABS: Vineland Adaptive Behavior Scale
SCQ: Social Communication Questionnaire
MAD: Median absolute deviation
