# Publisher Correction: Evaluation of familial phenotype deviation to measure the impact of de novo mutations in autism

**DOI:** 10.1186/s13073-025-01540-7

**Published:** 2025-09-04

**Authors:** Soo-Whee Kim, Hyeji Lee, Da Yea Song, Gang-Hee Lee, Jae Hyun Han, Jee Won Lee, Hee Jung Byun, Ji Hyun Son, Ye Rim Kim, Yoojeong Lee, Eunjoon Kim, Donna M. Werling, So Hyun Kim, Stephan J. Sanders, Hee Jeong Yoo, Joon-Yong An

**Affiliations:** 1 Department of Integrated Biomedical and Life Science, Korea University, Seoul, Republic of Korea; 2 L-HOPE Program for Community-Based Total Learning Health Systems, Korea University, Seoul, Republic of Korea; 3 Department of Psychiatry, Seoul National University Bundang Hospital, Seongnam, Republic of Korea; 4 Department of Psychiatry, Seoul National University College of Medicine, Seoul, Republic of Korea; 5 Department of Psychiatry, College of Medicine, Soonchunhyang University Cheonan Hospital, Cheonan, Republic of Korea; 6 Department of Psychiatry, Soonchunhyang University College of Medicine, Cheonan, Republic of Korea; 7 Department of Psychiatry, Seoul Metropolitan Children’s Hospital, Seoul, Republic of Korea; 8 Department of Biological Sciences, Korea Advanced Institute of Science and Technology, Daejeon, Republic of Korea; 9 Center for Synaptic Brain Dysfunctions, Institute for Basic Science, Daejeon, Republic of Korea; 10 Laboratory of Genetics, University of Wisconsin-Madison, Madison, WI USA; 11 Department of Psychology, Korea University, Seoul, Republic of Korea; 12 Institute of Developmental and Regenerative Medicine, Department of Pediatrics, University of Oxford, Oxford, UK; 13 Department of Psychiatry and Behavioral Sciences, UCSF Weill Institute for Neurosciences, University of California, San Francisco, USA; 14 School of Biosystem and Biomedical Science, College of Health Science, Korea University, Seoul, Republic of Korea

**Correction: Genome Medicine 17, 93 (2025)**

**https://doi.org/10.1186/s13073-025-01532-7**

Due to an error in the publication process the original publication of this article was missing the correspondence indication for Hee Jeong Yoo. The corresponding information is shown in this article, the original article has been updated. The publisher apologizes for the inconvenience caused to the authors & readers.

