# Supplementary material for: Evaluation of familial phenotype deviation to measure the impact of de novo mutations in autism
Source: Genome Med. 2025 Aug 20;17:93. doi: 10.1186/s13073-025-01532-7 (PMC12366145; doi:10.1186/s13073-025-01532-7)
Supplement: Supplementary file 1 — Additional file 1: 17 supporting Figures S1-S17. Captions for each Fig. S1 to S17 are given within the file. [file 13073_2025_1532_MOESM1_ESM.docx]

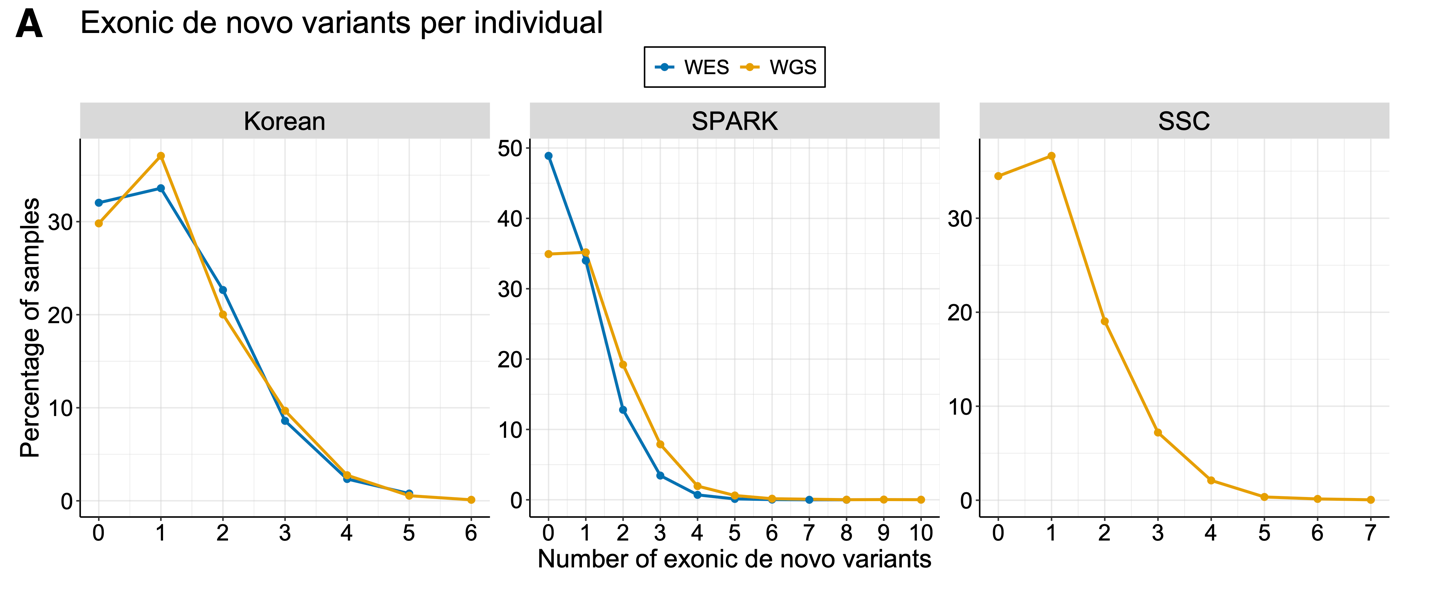


**Fig. S1| Distribution of exonic de novo variants per individual across cohorts and sequencing platforms**

**A,** Percentage of individuals with varying numbers of exonic de novo variants in the Korean, SPARK, and SSC cohorts, stratified by sequencing platform (WES vs. WGS).

**
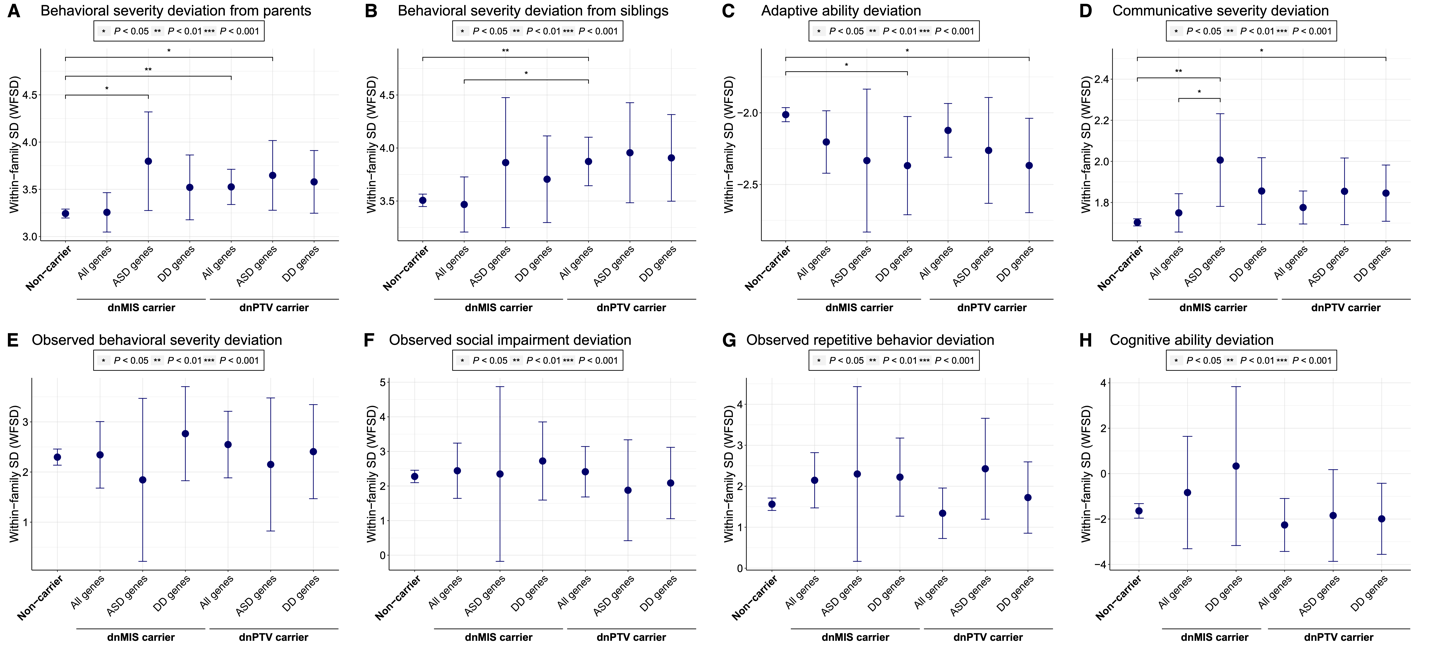
**

**Fig. S2| Comparison of intrafamilial deviations**

**A-D,** Within-family standard deviation (WFSD) of ASD probands stratified by genetic subgroup including non-carriers, de novo missense (dnMIS) carriers, and de novo protein-truncating variant (dnPTV) carriers; **A,** Behavioral symptom severity from parents; **B,** Behavioral symptom severity from unaffected siblings; **C,** Adaptive ability from unaffected siblings; **D,** Communicative symptom severity from unaffected siblings; **E,** Observed behavioral severity from unaffected siblings; **F,** Observed social impairments from unaffected siblings; **G,** Observed repetitive behaviors from unaffected siblings; **H,** Cognitive ability from unaffected siblings.


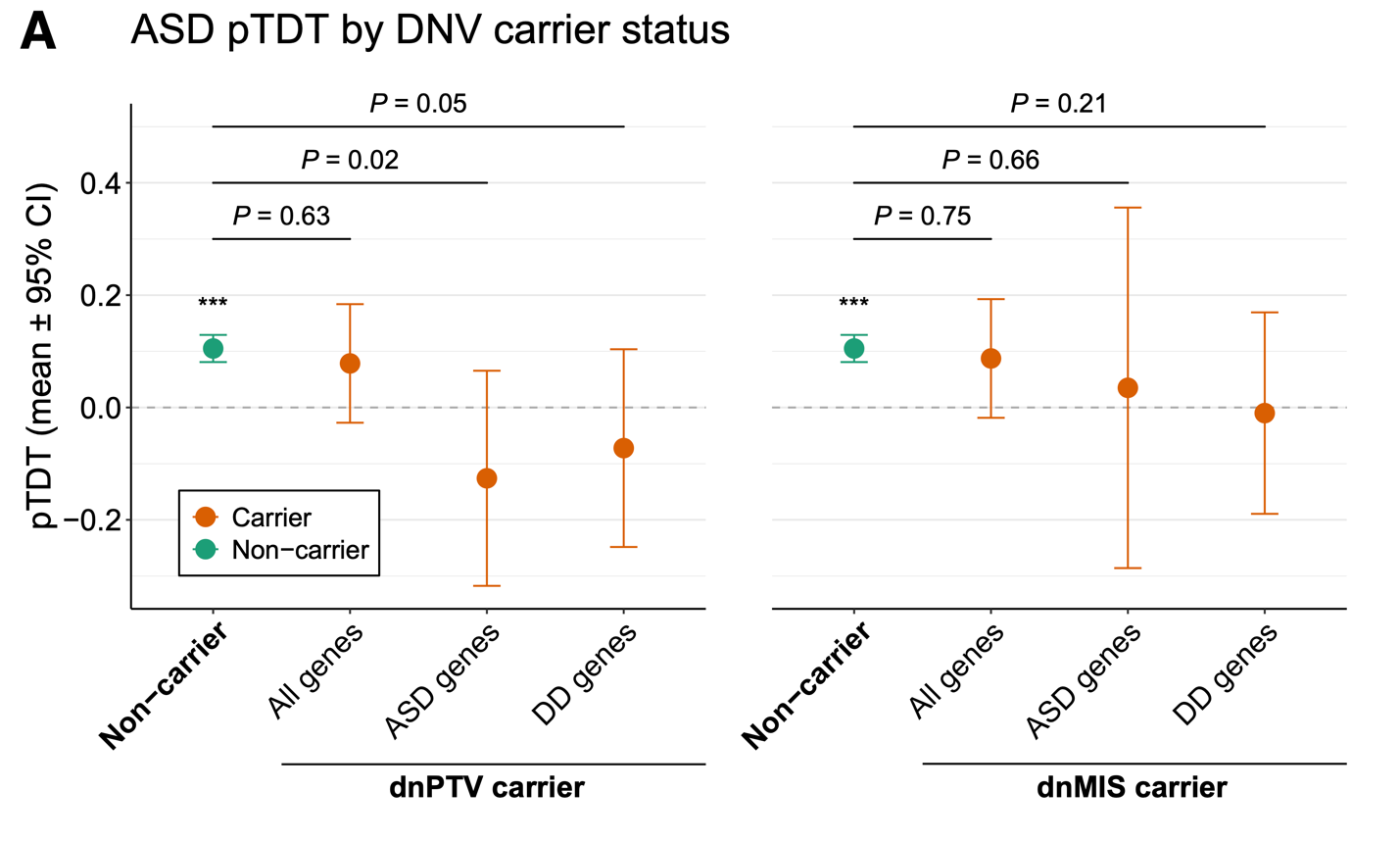


**Fig. S3| Polygenic transmission disequilibrium (pTDT) by DNV carrier status**

**A,** pTDT scores for ASD polygenic risk were compared between *de novo* missense (dnMIS) and *de novo* protein-truncating variant (dnPTV) carriers and non-carriers. Left, dnPTV carriers; right, dnMIS carriers. Each panel shows pTDT means (± 95% CI) for probands with DNVs in all genes, ASD-associated genes, or DD-associated genes, compared to non-carriers. Asterisks indicate deviation from zero tested using a one-sample t-test (“***”, *P* < 0.001; “**”, *P* < 0.01; “*”, *P* < 0.05). Horizontal *P*-values indicate group differences tested using a two-sample t-test.

**
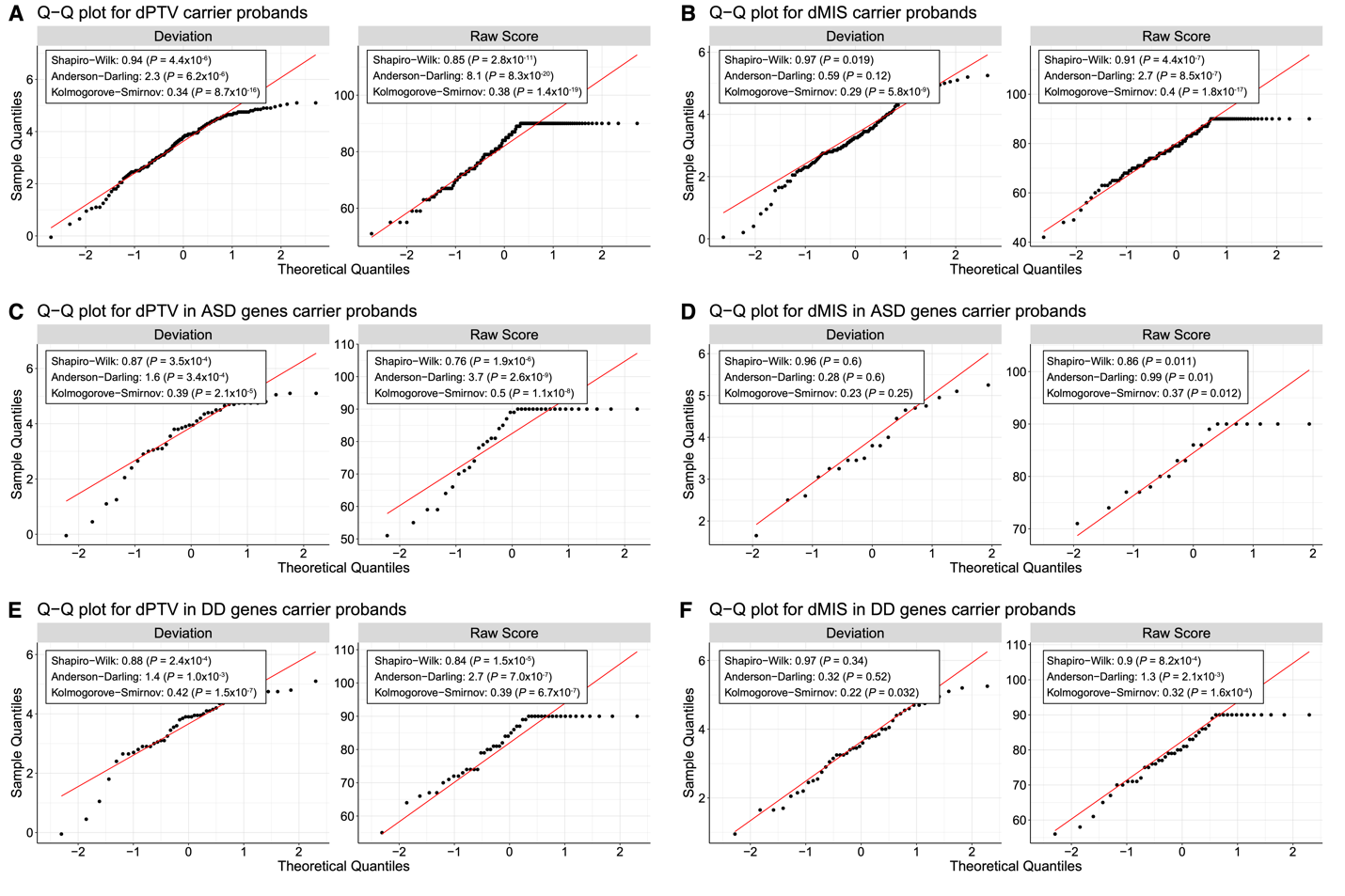
**

**Fig. S4| Distribution of raw phenotype scores and intrafamilial deviations from parents**

**A-F,** Quantile-quantile (Q-Q) plots of raw social responsiveness scale (SRS) T-scores and intrafamilial deviations from parents in ASD probands with; **A,** *de novo* protein-truncating variants (dnPTV); **B,** *de novo* missense (dnMIS) variants; **C,** dnPTV in ASD-associated genes; **D,** dnMIS in ASD-associated genes; **E,** dnPTV in developmental disorder (DD)-associated genes; **F,** dnMIS in DD-associated genes.


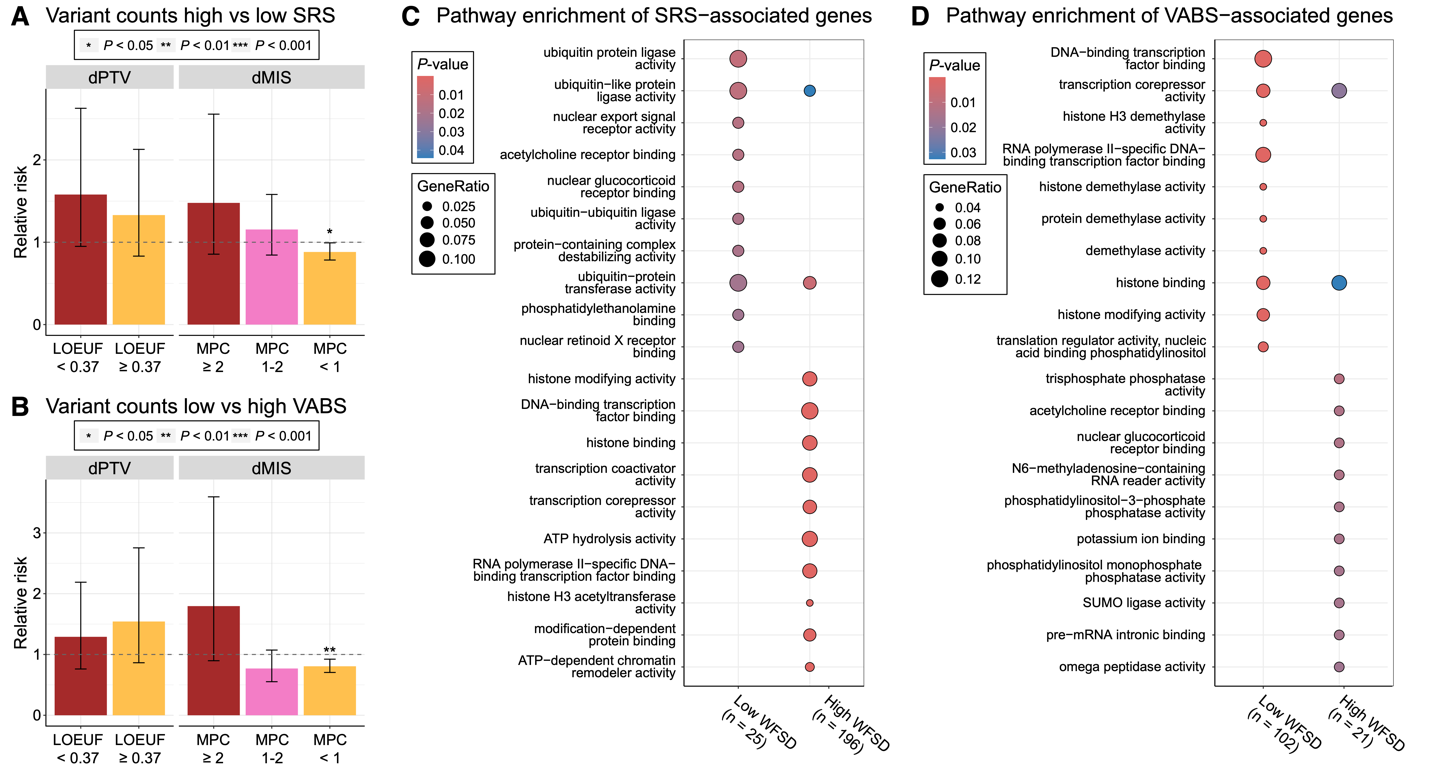


**Fig. S5| Variant type enrichment and pathway analysis in individuals with extreme phenotypic deviations**

**A,** Relative risk of carrying specific de novo variant types in individuals with the severe subgroup (high; SRS WFSD ≥ 2.6) vs. better-than-expected subgroup (low; SRS WFSD < 2.0) social responsiveness deviation. **B,** Relative risk in individuals with the severe (low; VABS WFSD ≤ -2) vs. better-than-expected subgroup (high; VABS WFSD > -1) adaptive functioning deviation. Error bars represent 95% confidence intervals. Significance levels are denoted by asterisks (‘***’, *P* < 0.001; ‘**’, *P* < 0.01; ‘*’, *P* < 0.05). **C,** Gene ontology (GO) enrichment analysis of genes carrying LOEUF < 0.37 dnPTV or MPC ≥ 2 dnMIS variants in each SRS subgroup. **D,** GO enrichment for the same gene sets in each VABS subgroup. Point size indicates the proportion of input genes annotated to each term (GeneRatio), and color indicates *P*-value.

**
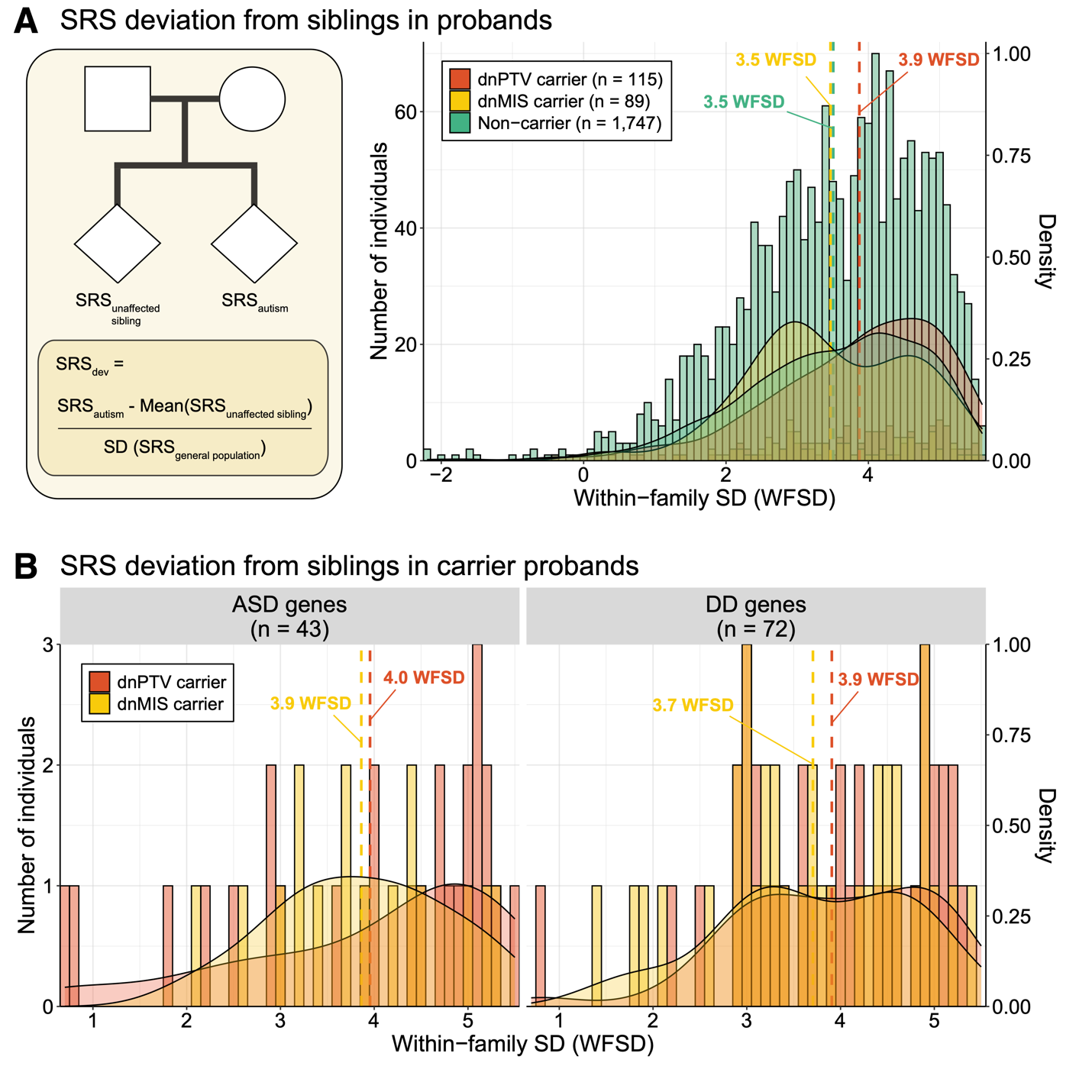
**

**Fig. S6| Intrafamilial deviation of** **social responsiveness scale from unaffected siblings**

**A,** Within-family standard deviation (WFSD) calculation for social responsiveness scale (SRS) in ASD cases based on unaffected siblings: subtracting mean sibling SRS T-scores and normalizing by the general population SD. Histogram shows SRS deviation distributions for probands (n = 1,951) by genetic subgroup. **B,** Stratification of SRS deviations by ASD- and developmental disorder (DD)-associated gene sets.

**
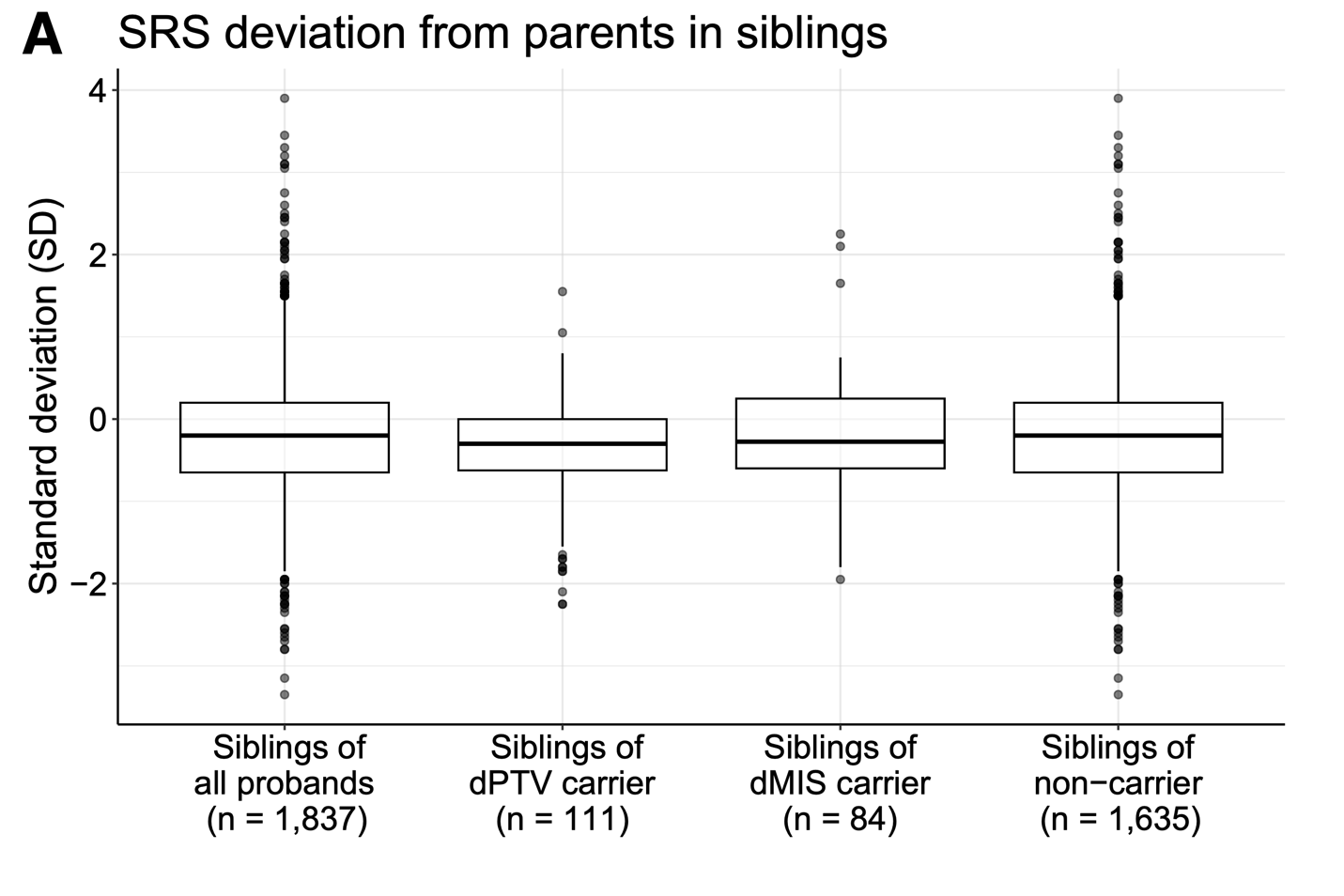
**

**Fig. S7| Intrafamilial deviation of social responsiveness scale from parents in unaffected siblings**

**A,** Distribution of social responsiveness scale (SRS) within-family standard deviation (WFSD) in unaffected siblings (n = 1,837), stratified by the genetic subgroup of ASD probands within the same family.

**
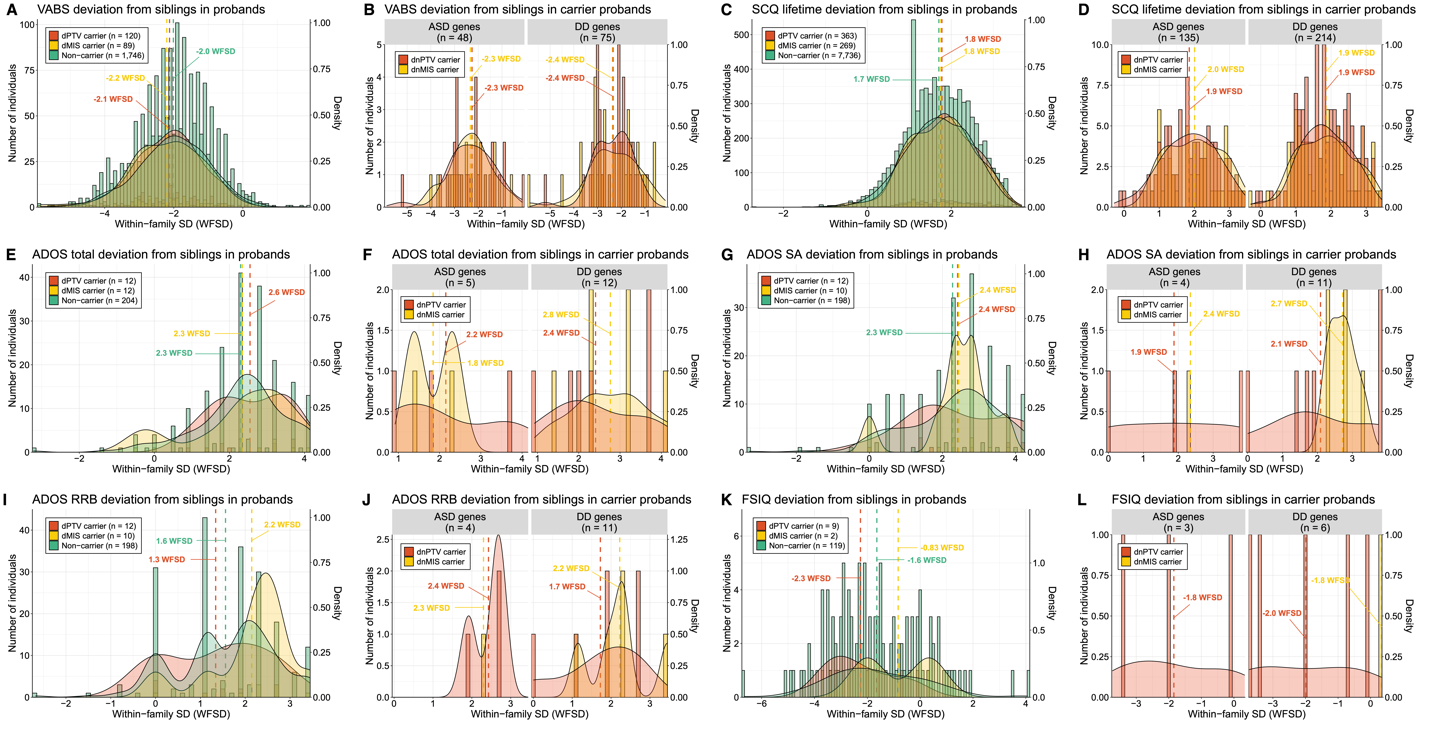
**

**Fig. S8| Intrafamilial deviation of** **Vineland adaptive behavior scale and** **social communication questionnaire lifetime from unaffected siblings**

Within-family standard deviation (WFSD) of: **A,** Vineland adaptive behavior scale (VABS), **C,** social communication questionnaire (SCQ) lifetime, **E,** ADOS calibrated severity scores (CSS) total, **G,** ADOS CSS social affect (SA), **I,** ADOS CSS restricted/repetitive behavior (RRB), and **K,** full-scale IQ (FSIQ) in ASD cases from unaffected siblings depending on genetic subgroups and **B,** VABS, **D,** SCQ lifetime, **F,** ADOS total, **H,** ADOS SA, **J,** ADOS RRB, and **L,** FSIQ deviations by ASD- and developmental disorder (DD)-associated genes.

**
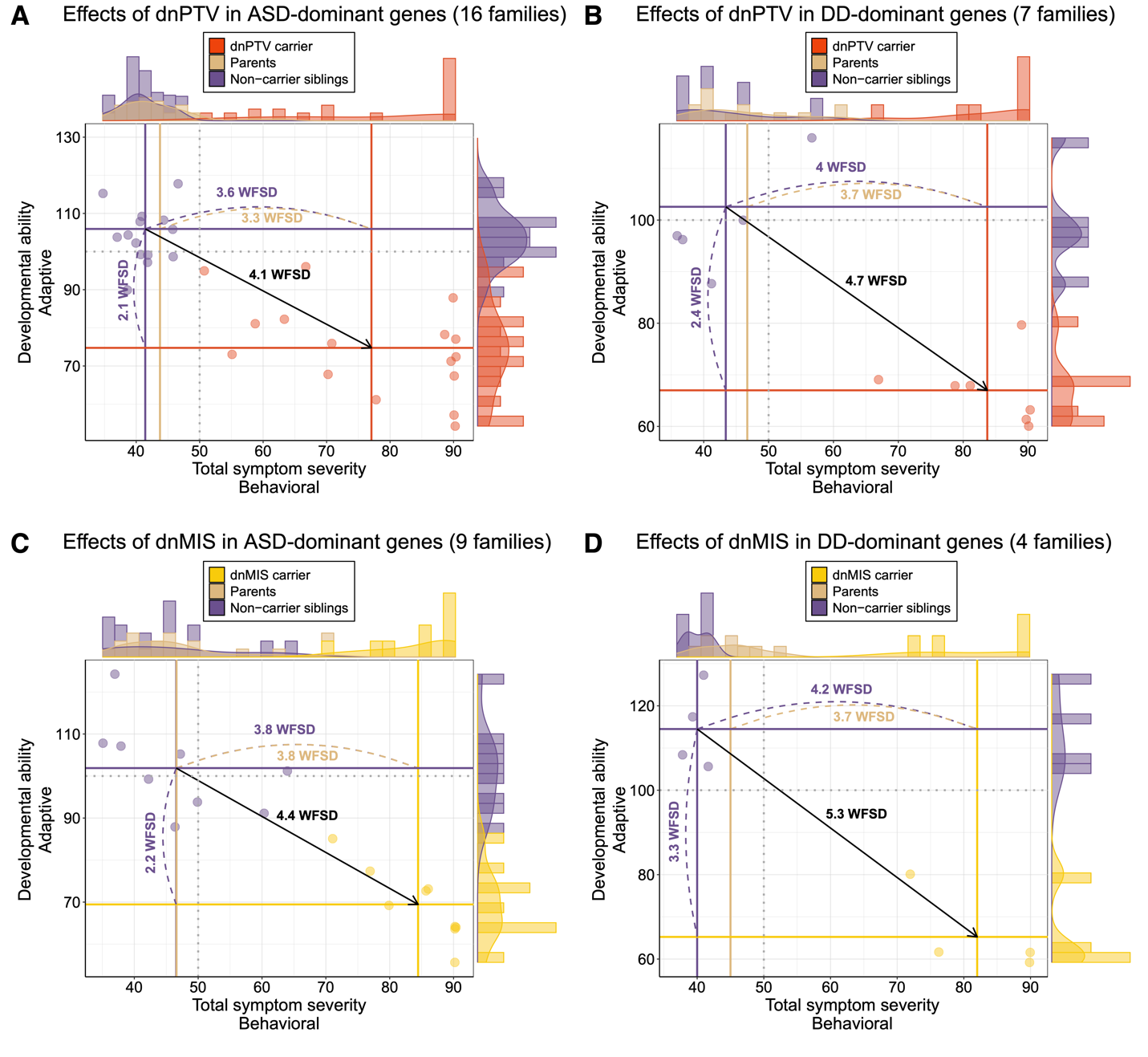
**

**Fig. S9| Effects of *de novo* variants on neurodevelopmental profiles**

Neurodevelopmental profile deviation in 2D space for: **A,** *de novo* protein-truncating variant (dnPTV) in ASD-dominant genes (n = 16 families); **B,** dnPTV in developmental disorder (DD)-dominant genes (n = 7 families); **C,** *de novo* missense (dnMIS) in ASD-dominant genes (n = 9 families); **D,** dnMIS in DD-dominant genes (n = 4 families).


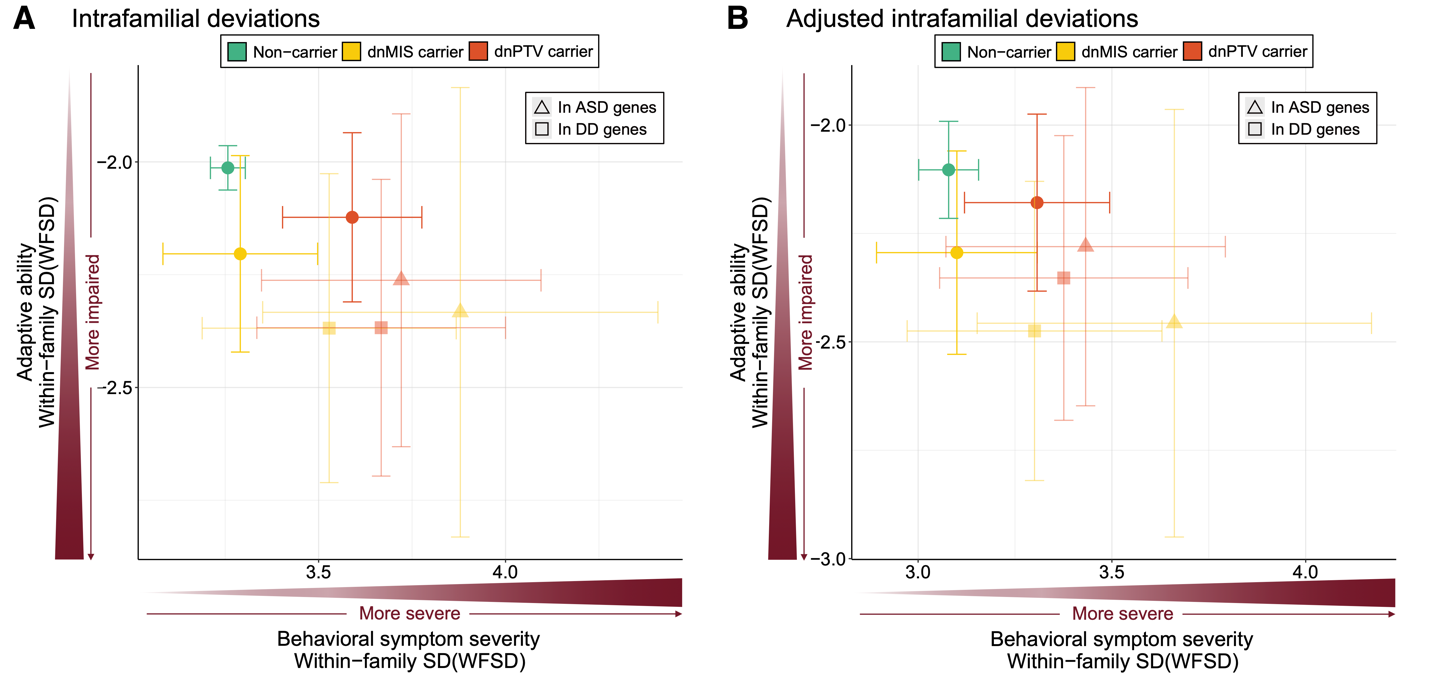


**Fig. S10| Intrafamilial deviations of total symptom severity and developmental ability**

Within-family standard deviation (WFSD) for total symptom severity (behavioral: SRS; higher, more severe) and developmental ability (adaptive ability: VABS total; lower, more impaired) in ASD probands depending by genetic subgroup: **A,** Unadjusted and **B,** Adjusted for age, sex, and the cohort by conducting generalized linear model regression.


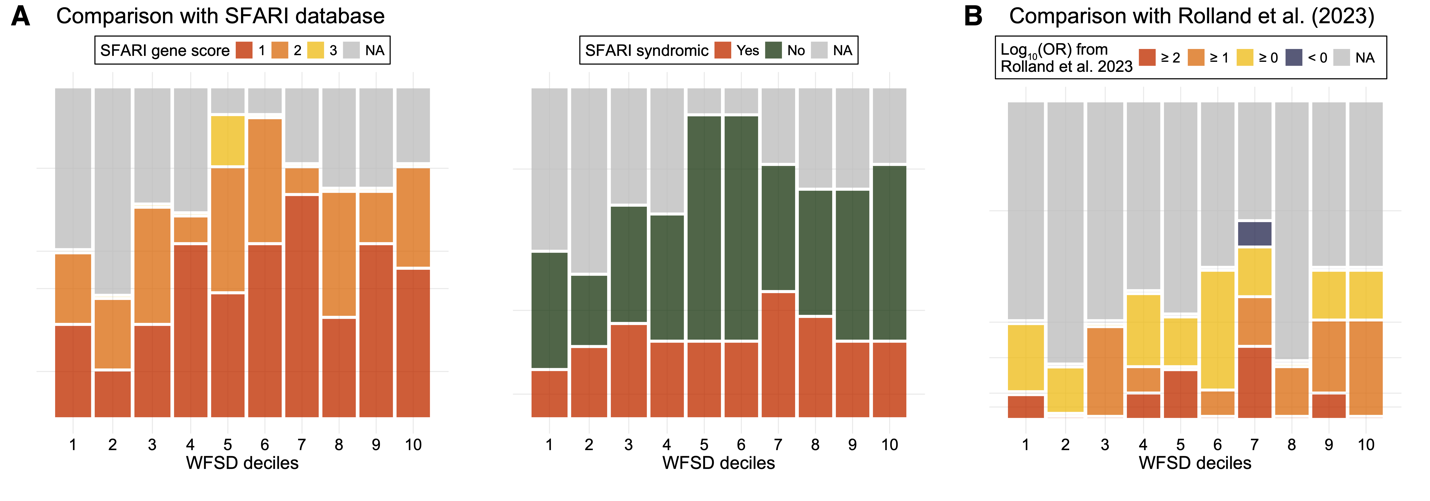


**Fig. S11| Comparison of gene-level effects of dnPTV on SRS WFSD with external gene annotations and effect size estimates**

**A,** Distribution of SFARI Gene scores (1–3) and syndromic gene annotations across deciles of mean WFSD SRS scores for dnPTV carriers in ASD probands. **C,** Distribution of Log_10_ odds ratio (OR) categories from Rolland et al. across WFSD deciles. WFSD deciles were computed by ranking genes based on the mean within-family standardized SRS deviation among dnPTV carriers.


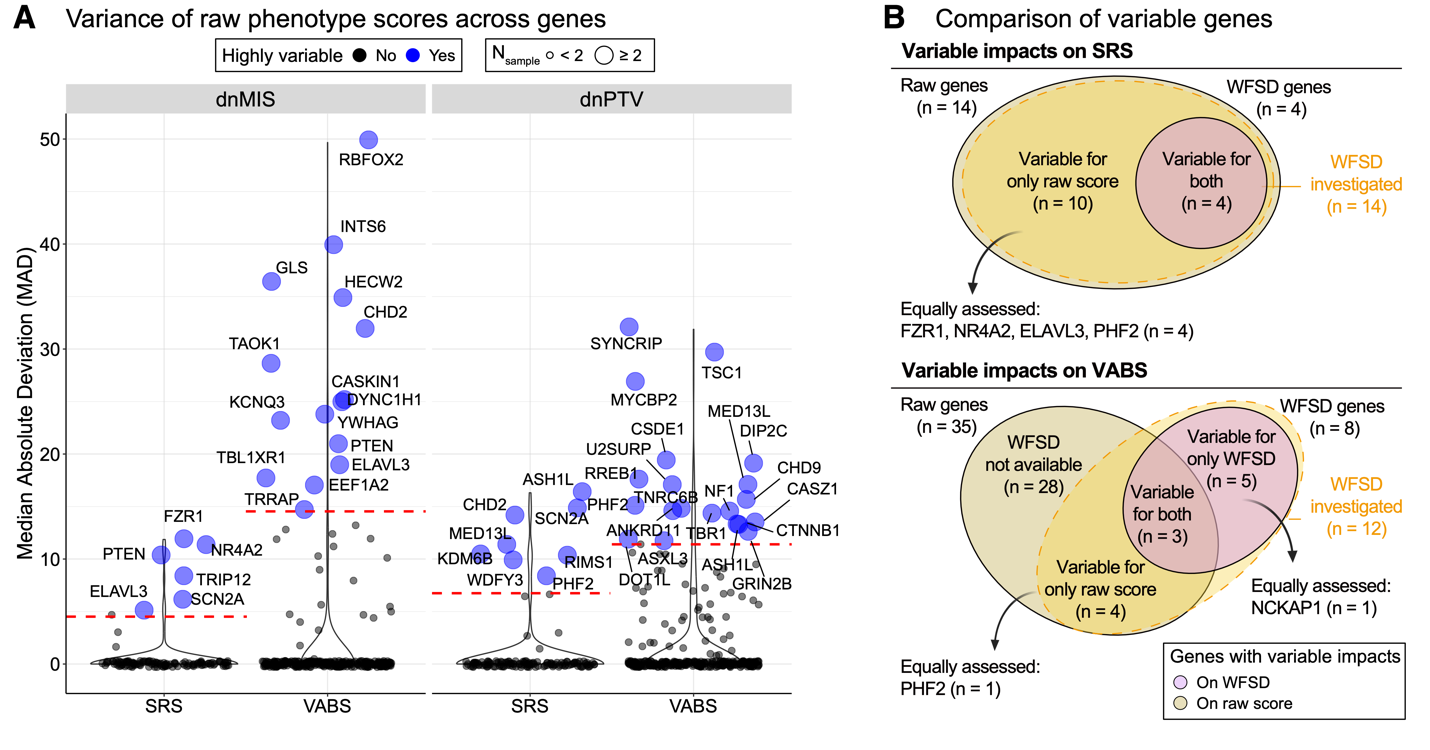


**Fig. S12| Genes with variable expressivity between individuals for raw phenotype scores**

**A,** Median absolute deviation (MAD) of phenotype outcomes of *de novo* missense (dnMIS) and protein-truncating variant (dnPTV) for each gene, highlighting outlier genes with highly variable impacts on raw phenotype scores between unrelated individuals. **B,** Comparison of genes with variable impacts between families on within-family standard deviation (WFSD) and on raw score. Among equally assessed genes (where the same number of probands – carrying DNV in the gene – is available for WFSD and raw score), *NCKAP1* was exclusively identified using WFSD to have variable impacts on Vineland adaptive behavior scale (VABS). On the other hand, 4 genes were exclusively identified using raw scores to have variable impacts on social responsiveness scale (SRS) T-scores and one gene on VABS score.


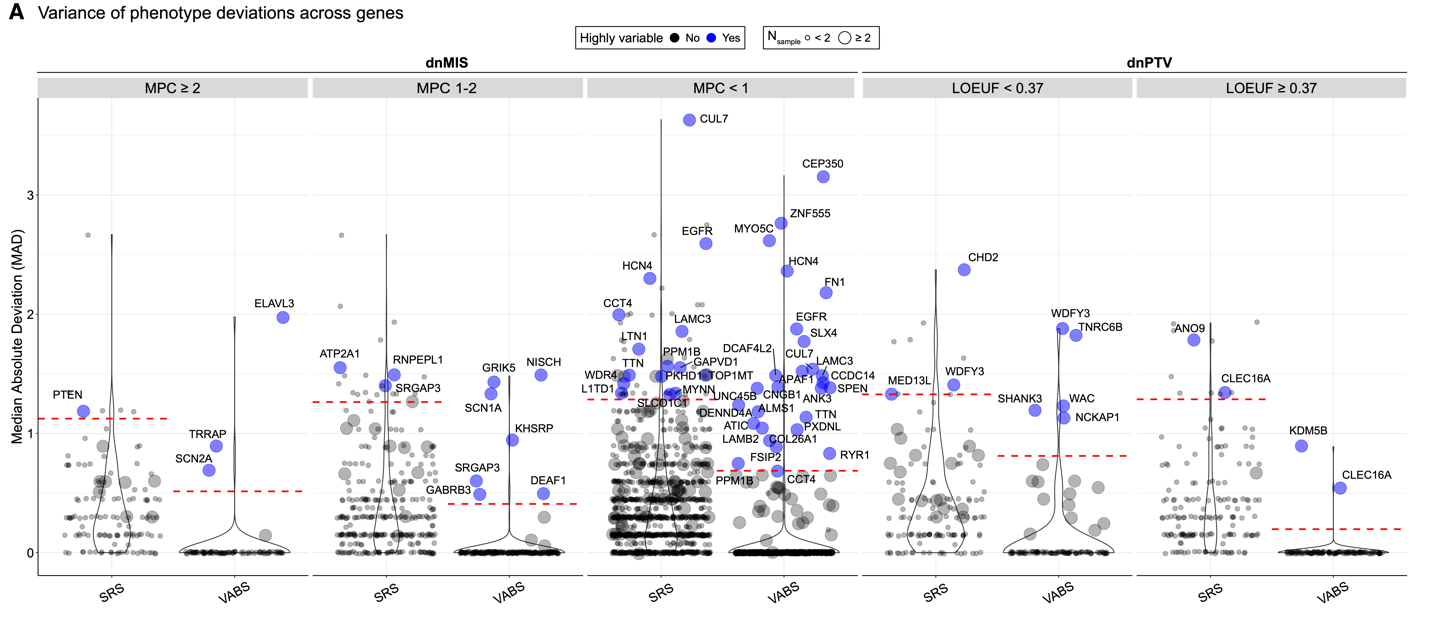


**Fig. S13| Genes with variable expressivity between families for neurodevelopmental profiles**

**A,** Median absolute deviation (MAD) of intrafamilial deviations of *de novo* missense (dnMIS) across missense badness, PolyPhen-2, constraint (MPC) scores and protein-truncating variant (dnPTV) across loss-of-function observed over expected upper bound fraction (LOEUF) scores for each gene.


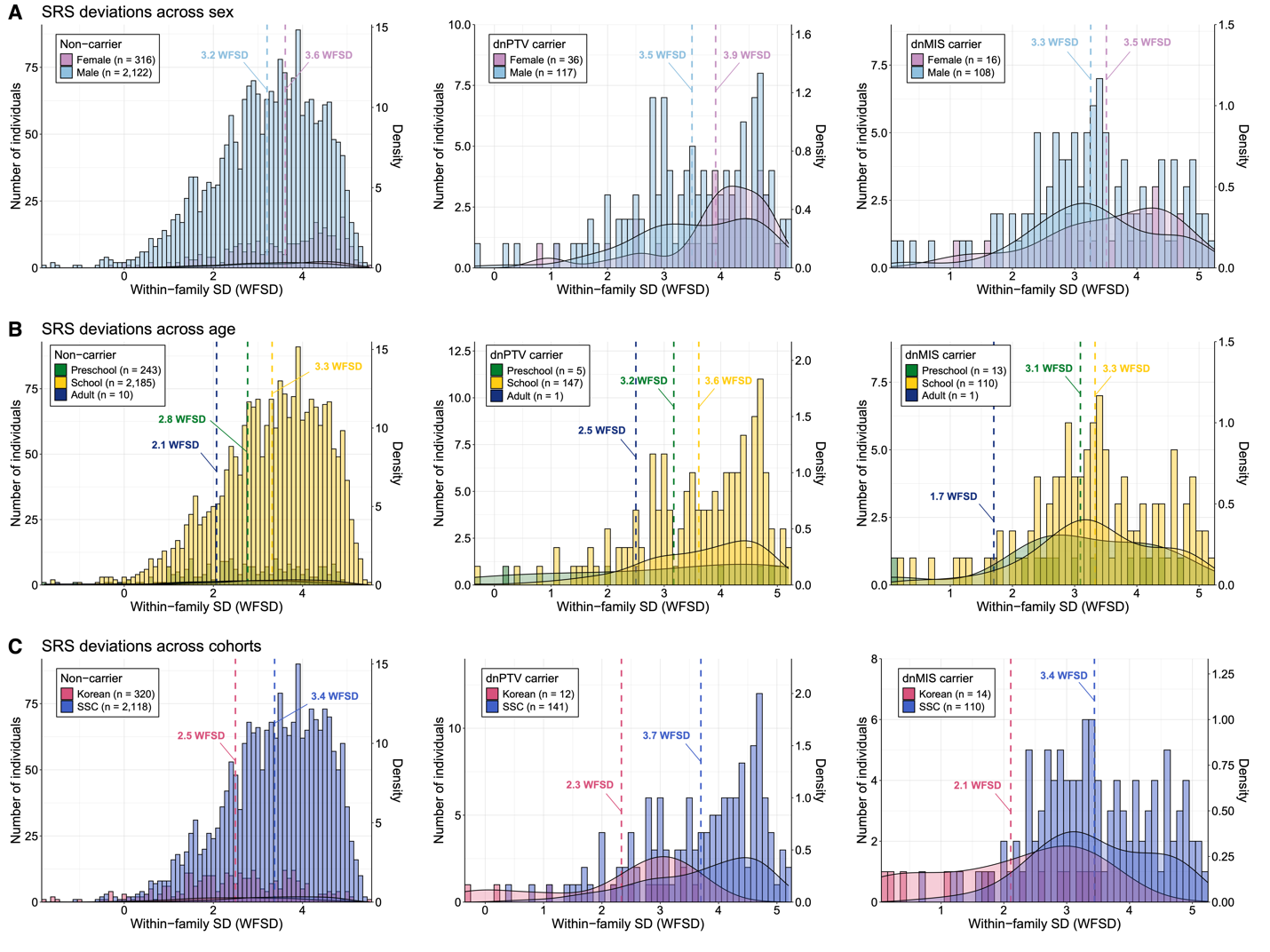


**Fig. S14| Intrafamilial deviations of social responsive scale across sex, age, and cohorts**

Social responsiveness scale (SRS) deviations in non-carriers, *de novo* protein-truncating variant (dnPTV) carriers, and missense (dnMIS) carriers across; **A,** sex; **B,** age (preschool: ≤ 4.5 years; school: 4.5-19 years; adult: > 19 years); **C,** cohorts.


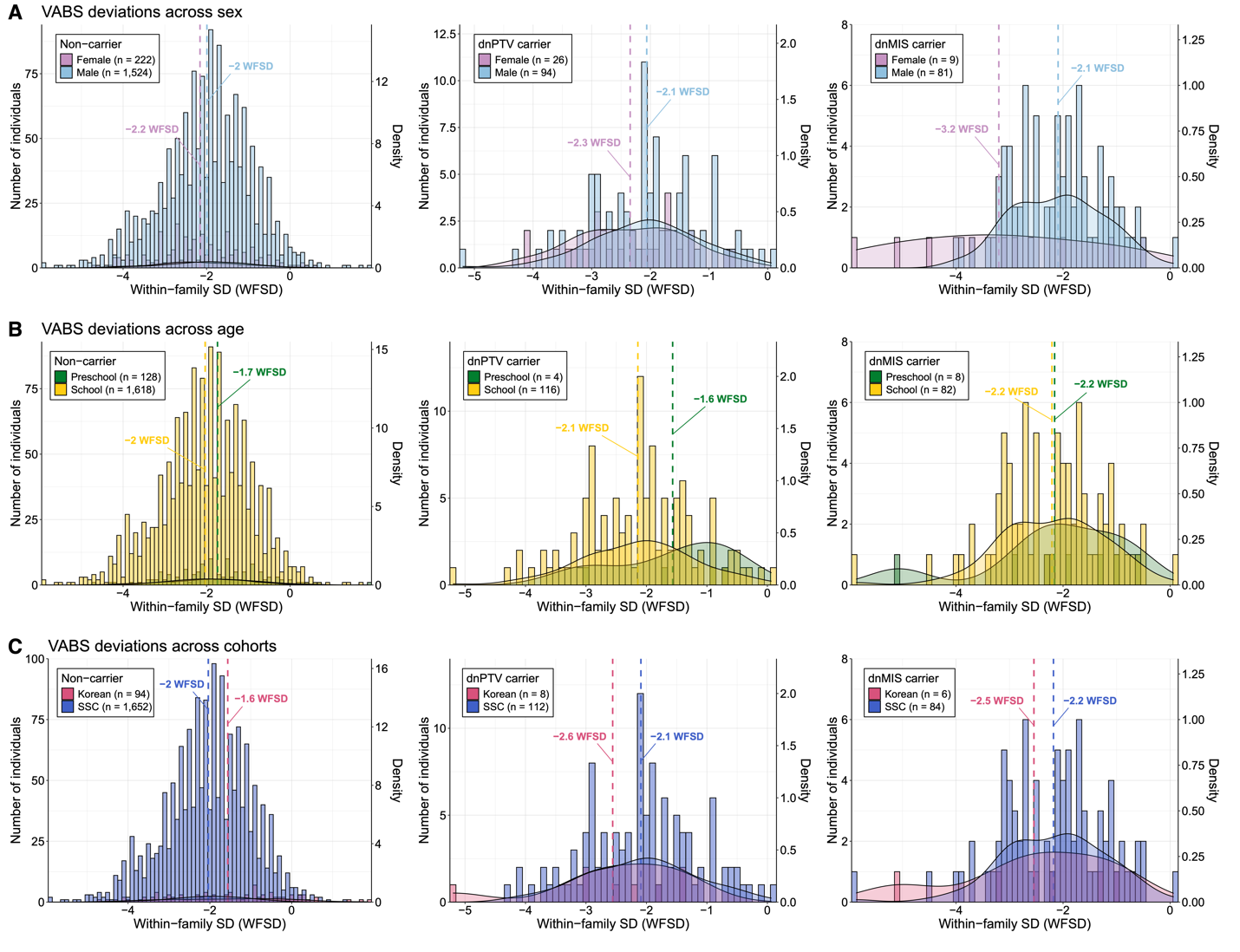


**Fig. S15| Intrafamilial deviations of Vineland adaptive behavior scale across sex, age, and cohorts**

Vineland adaptive behavior scale (VABS) deviations in non-carriers, *de novo* protein-truncating variant (dnPTV) carriers, and missense (dnMIS) carriers across; **A,** sex; **B,** age (preschool: ≤ 4.5 years; school: 4.5-19 years); **C,** cohorts.


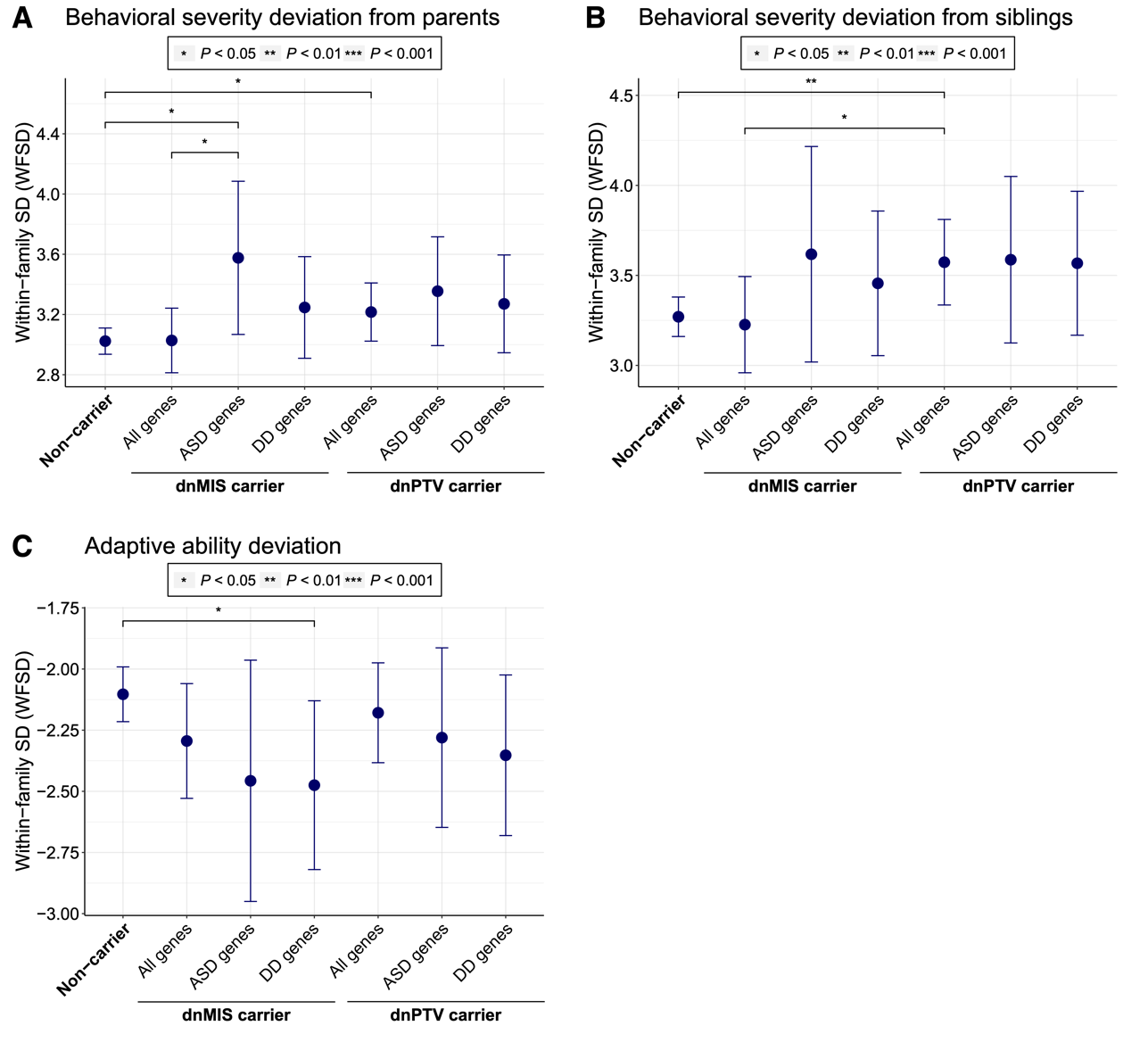


**Fig. S16| Comparison of intrafamilial deviations adjusted for sex, age, and cohorts**

**A-C,** Within-family SD (WFSD) in ASD probands adjusted for sex, age, and cohorts across genetic subgroups including non-carriers, *de novo* missense (dnMIS) carriers, and *de novo* protein-truncating variant (dnPTV) carriers; **A,** behavioral symptom severity from parents; **B,** behavioral symptom severity from unaffected siblings; **C,** adaptive ability from unaffected siblings.


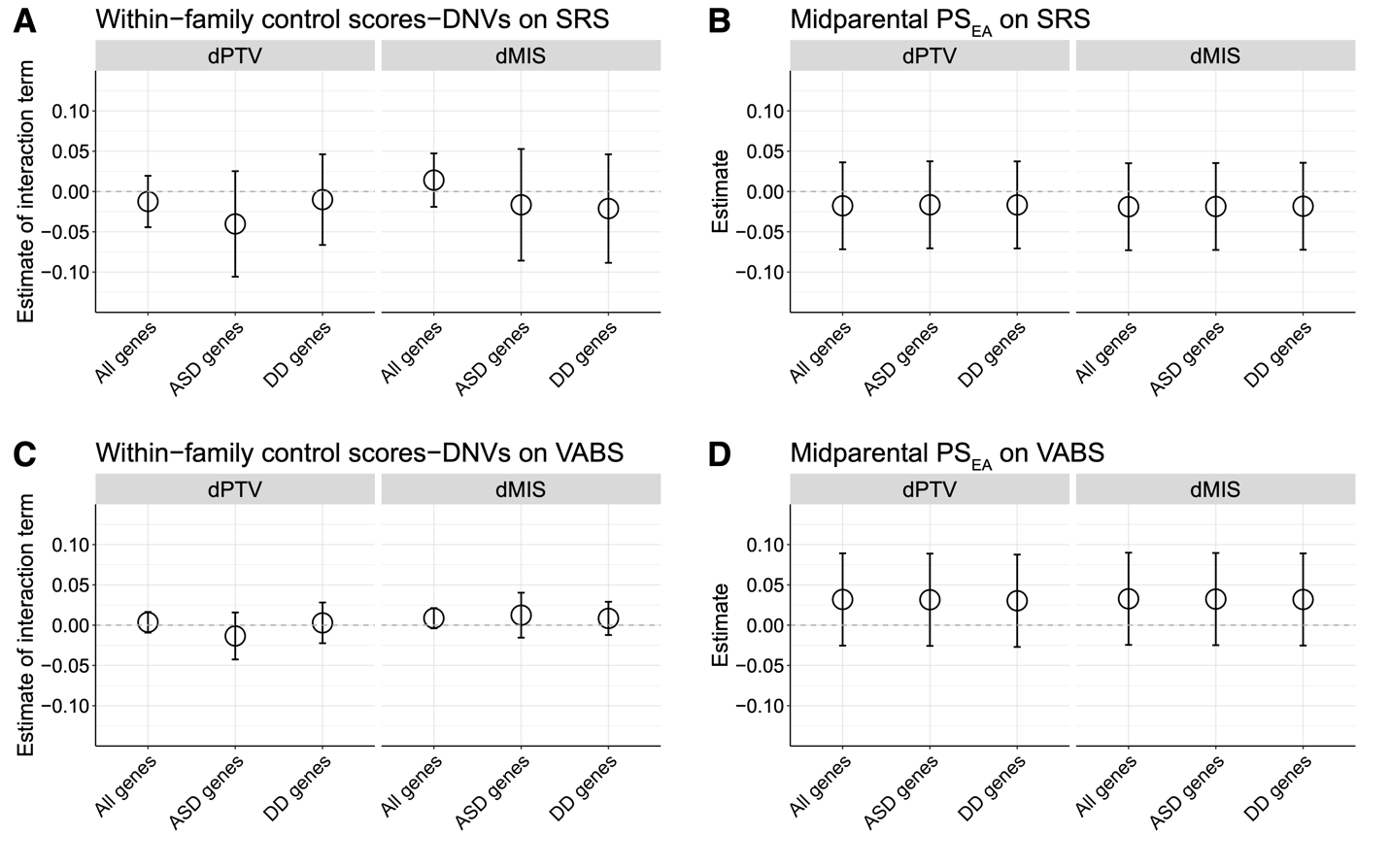


**Fig. S17| Interaction analyses assessing potential ascertainment bias in WFSD estimates**

**A,** Estimated interaction effects between within-family control SRS scores and DNV carrier status on SRS WFSD. **B,** Estimated interaction effects between midparental educational attainment polygenic scores (PS_EA_) and DNV carrier status on SRS WFSD. **C,** Estimated interaction effects between within-family control VABS scores and DNV carrier status on VABS WFSD. **D,** Estimated interaction effects between midparental PSEA and DNV carrier status on VABS WFSD. Error bars represent 95% confidence intervals. All estimates were non-significant; open circles indicate no significant interaction effect (*P* > 0.05).
